# Antidiabetic Potential of Medicinal Plants and Their Active Components

**DOI:** 10.3390/biom9100551

**Published:** 2019-09-30

**Authors:** Bahare Salehi, Athar Ata, Nanjangud V. Anil Kumar, Farukh Sharopov, Karina Ramírez-Alarcón, Ana Ruiz-Ortega, Seyed Abdulmajid Ayatollahi, Patrick Valere Tsouh Fokou, Farzad Kobarfard, Zainul Amiruddin Zakaria, Marcello Iriti, Yasaman Taheri, Miquel Martorell, Antoni Sureda, William N. Setzer, Alessandra Durazzo, Massimo Lucarini, Antonello Santini, Raffaele Capasso, Elise Adrian Ostrander, Atta -ur-Rahman, Muhammad Iqbal Choudhary, William C. Cho, Javad Sharifi-Rad

**Affiliations:** 1 Student Research Committee, School of Medicine, Bam University of Medical Sciences, Bam 44340847, Iran; bahar.salehi007@gmail.com; 2 Department of Chemistry, Richardson College for the Environmental Science Complex, The University of Winnipeg, Winnipeg, MB R3B 2G3, Canada; a.ata@uwinnipeg.ca; 3 Department of Chemistry, Manipal Institute of Technology, Manipal University, Manipal 576104, India; nv.anil@manipal.edu; 4 Department of Pharmaceutical Technology, Avicenna Tajik State Medical University, Rudaki 139, Dushanbe 734003, Tajikistan; shfarukh@mail.ru; 5 Department of Nutrition and Dietetics, Faculty of Pharmacy, University of Concepcion, Concepción 4070386, Chile; karramir@gmail.com; 6 Facultad de Educación y Ciencias Sociales, Universidad Andrés Bello, Autopista Concepción—Talcahuano, Concepción 7100, Chile; a.ruizortega@uandresbello.edu; 7 Phytochemistry Research Center, Shahid Beheshti University of Medical Sciences, Tehran 1991953381, Iran; majid_ayatollahi@yahoo.com (S.A.A.); farzadkf@yahoo.com (F.K.); taaheri.yasaman@gmail.com (Y.T.); 8 Department of Pharmacognosy, School of Pharmacy, Shahid Beheshti University of Medical Sciences, Tehran 11369, Iran; 9 Department of Biochemistry, Faculty of Science, University of Yaounde 1, Yaounde P.O. Box 812, Cameroon; ptsouh@gmail.com; 10 Department of Medicinal Chemistry, School of Pharmacy, Shahid Beheshti University of Medical Sciences, Tehran 11369, Iran; 11 Laboratory of Halal Science Research, Halal Products Research Institute, Universiti Putra Malaysia, 43400 UPM Serdang, Selangor, Malaysia; dr_zaz@yahoo.com; 12 Integrative Pharmacogenomics Institute (iPROMISE), Faculty of Pharmacy, Universiti Teknologi MARA, Puncak Alam Campus, Bandar Puncak Alam Selangor 42300, Malaysia; 13 Department of Agricultural and Environmental Sciences, Milan State University, via G. Celoria 2, 20133 Milan, Italy; 14 Universidad de Concepción, Unidad de Desarrollo Tecnológico, UDT, Concepción 4070386, Chile; 15 Research Group on Community Nutrition and Oxidative Stress, Laboratory of Physical Activity Sciences, and CIBEROBN—Physiopathology of Obesity and Nutrition, CB12/03/30038, University of Balearic Islands, E-07122 Palma de Mallorca, Spain; tosugo@hotmail.com; 16 Department of Chemistry, University of Alabama in Huntsville, Huntsville, AL 35899, USA; setzerw@uah.edu; 17 CREA—Research Centre for Food and Nutrition, Via Ardeatina 546, 00178 Rome, Italy; alessandra.durazzo@crea.gov.it (A.D.); massimo.lucarini@crea.gov.it (M.L.); 18 Department of Pharmacy, University of Napoli Federico II, Via D. Montesano, 49-80131 Napoli, Italy; 19 Department of Agricultural Sciences, University of Naples Federico II, 80055 Portici, Italy; rafcapas@unina.it; 20 Medical Illustration, Kendall College of Art and Design, Ferris State University, Grand Rapids, MI 49503, USA; eliseadrianostrander@gmail.com; 21 H.E.J. Research Institute of Chemistry, International Center for Chemical and Biological Sciences, University of Karachi, Karachi 75270, Pakistan; aurahman786@gmail.com (A.-u.-R.); iqbal.choudhary@iccs.edu (M.I.C.); 22 Department of Clinical Oncology, Queen Elizabeth Hospital, Kowloon, Hong Kong, China; 23 Department of Pharmacology, Faculty of Medicine, Jiroft University of Medical Sciences, Jiroft 7861756447, Iran

**Keywords:** Diabetes mellitus, medicinal plants, antidiabetic, hypoglycemic, antihyperglycemic

## Abstract

Diabetes mellitus is one of the major health problems in the world, the incidence and associated mortality are increasing. Inadequate regulation of the blood sugar imposes serious consequences for health. Conventional antidiabetic drugs are effective, however, also with unavoidable side effects. On the other hand, medicinal plants may act as an alternative source of antidiabetic agents. Examples of medicinal plants with antidiabetic potential are described, with focuses on preclinical and clinical studies. The beneficial potential of each plant matrix is given by the combined and concerted action of their profile of biologically active compounds.

## 1. Introduction

Diabetes mellitus (DM) is a serious, chronic, and complex metabolic disorder of multiple aetiologies with profound consequences, both acute and chronic [1]. Also known only as diabetes, DM and its complications affect people both in the developing and developed countries, leading to a major socioeconomic challenge. It is estimated that 25% of the world population is affected by this disease [2]. Genetic and environmental factors contribute significantly to the development of diabetes [3]. During the development of diabetes, the cells of the body cannot metabolize sugar properly due to deficient action of insulin on target tissues resulting from insensitivity or lack of insulin (a peptide hormone that regulates blood glucose). The inability of insulin to metabolize sugar occurs when the pancreas does not produce enough insulin or when the body cannot effectively use the insulin it produces. This triggers the body to break down its own fat, protein, and glycogen to produce sugar, leading to the presence of high sugar levels in the blood with excess by-products called ketones being produced by the liver [4,5]. Diabetes is distinguished by chronic hyperglycemia with disturbances in the macromolecules’ metabolism as a result of impairments in insulin secretion, insulin action, or both. Diabetes causes long-term damage, dysfunction, and failure of various organ systems (heart, blood vessels, eyes, kidneys, and nerves), leading to disability and premature death [6]. The severity of damage triggered by hyperglycemia on the respective organ systems may be related to how long the disease has been present and how well it has been controlled. Several symptoms such as thirst, polyuria, blurring of vision, and weight loss also accompany diabetes [7].

## 2. Types of Diabetes, Prevalence, and Management

There are various types of diabetes of which type 1 DM (T1DM) and type 2 DM (T2DM) were the most usually discussed. The T1DM is also known as insulin-dependent diabetes. It is primarily due to pancreatic islet beta cell destruction and is characterized by deficient insulin production in the body [6]. Patients with T1DM are prone to ketoacidosis and need daily administration of insulin to control the amount of glucose in their blood. The majority of T1DM occurs in children and adolescents [5]. On the other hand, T2DM, also known as non-insulin-dependent diabetes, results from the body’s ineffective use of insulin and hyperglycemia [8,9] and accounts for the vast majority of people with diabetes around the world. Insulin resistance is due to a reduced responsiveness of target tissues to normal circulating levels of insulin [9]. Ethnicity, family history of diabetes, and previous gestational diabetes, older age, overweight and obesity, unhealthy diet, physical inactivity, and smoking increase diabetes risk. Most people with diabetes are affected by T2DM diabetes (90%), usually occur nearly entirely among adults but, in these days, is increasing in children [5].

The universal prevalence of diabetes has nearly doubled since 1980, rising from 4.7% to 8.5% in the adult population. Moreover, the prevalence of diabetes has also been found to steadily increase for the past 3 decades and has risen faster in low- and middle-income countries compared to high-income countries. The increase in the prevalence of diabetes is parallel with an increase in associated risk factors such as being overweight or obese. If not properly treated or controlled, diabetes may cause blindness, kidney failure, lower limb amputation, and other long-term consequences that impact significantly on the quality of life [10]. Interestingly, the WHO also projects that diabetes will be the seventh leading cause of death in 2030 [11]. The incidence and prevalence of diabetes have continued to increase globally, despite a great deal of research with the resulting burden resting more heavily on tropical developing countries [12,13]. Based on demographic studies, by 2030, the number of people older than 64 years with diabetes will be greater in developing countries (≥82 million) in comparison to that in developed countries (≥48 million). The greatest relative increases are projected to occur in the Middle East crescent, sub-Saharan Africa, and India [14,15].

Amongst all people with diabetes, T2DM accounts for the majority (90%) of cases, and these can be prevented as well as treated easily, while T1DM cannot be prevented with current knowledge. Since management of diabetes is complex and multidisciplinary, it should include primary prevention through promotion of a healthy diet and lifestyle (such as exercise). Dietary management and exercise represent important pillars of care and are crucial in the treatment of T2DM, and both may be adequate to attain and retain the therapeutic goals to normolipidemic and normoglycemia.

## 3. Antidiabetic Drugs and Their Side Effects

There are several classes of oral hypoglycemic drugs that exert antidiabetic effects through different mechanisms, namely sulfonylureas, biguanides, α-glucosidase inhibitors, thiazolidinediones, and non-sulfonylureas secretagogues. Oral sulfonylureas, such as glimepiride and glyburide, act to reduce blood sugar, mainly by elevating insulin release from islets of Langerhans. This is achieved through binding with the sulfonylurea receptor on β cells resulting in adenosine triphosphate-dependent potassium channels closure. As a result, the cell membrane depolarizes and the following calcium influx accompanied by secretion of stored insulin from secretory granules within the cells takes place. This mechanism works only in the presence of insulin [16,17].

Another oral hypoglycemic drug, the biguanides, acts to reduce hepatic gluconeogenesis and to replenish peripheral tissues’ sensitivity to insulin, actions that are achieved through elevation of insulin-stimulated uptake and use of sugar. Nevertheless, biguanides are ineffective in insulin absence. The best example of this class is metformin.

The α-glucosidase inhibitors, such as acarbose and miglitol, impede certain enzymes responsible for the breakdown of carbohydrates in the small intestine. This class of hypoglycemic agents acts mostly by reducing the absorption rate of carbohydrates in the body. Also, acarbose reversibly inhibits both pancreatic α-amylase and α-glucosidase enzymes by binding to the carbohydrate-binding region and by interfering with their hydrolysis into monosaccharides, which leads to a slower absorption together with a reduction in postprandial blood sugar levels [16,18].

Another important class of oral hypoglycemic agents is the thiazolidinediones (TZDs), such as pioglitazone and rosiglitazone, of which the mechanism of action primarily includes improving muscle and adipose tissue sensitivity to insulin and, to a smaller extent, reducing liver glucose production. TZDs also are potent and selective agonists to the nuclear peroxisome proliferator-activated receptor gamma (PPARγ) present in liver, skeletal muscle, and adipose tissue. Activation of PPARγ receptors controls the transcription of insulin-responsive genes involved in the regulation of transportation, production, and glucose use. Also, TZDs have been reported to augment β-cell function by lowering free fatty acid levels that ultimately lead to β-cell death [19].

The last class of oral hypoglycemic agents is the non-sulfonylureas secretagogues, which include meglitinide and repaglinide and which increases the secretion of insulin from active β cells by a similar mechanism as sulfonylureas. However, this class of oral antidiabetic agents binds to different β-cell receptors [20].

Although synthetic oral hypoglycemic drugs alongside insulin are the main route for controlling diabetes, they fail to reverse the course of its complications completely and further worsen it by the fact that they also demonstrate prominent side effects. This forms the main force for discovering alternative sources of antidiabetic agents [21]. Despite the significant progress made in the treatment of diabetes using oral antidiabetic agents in the past three decades, the results of treatment of diabetic patients are still far from perfect. Several disadvantages have been reported related to the use of those oral hypoglycemic agents, including drug resistance (reduction of efficiency), adverse effects, and even toxicity. For example, sulfonylureas lose their effectiveness after 6 years of treatment in approximately 44% of patients, whereas glucose-lowering drugs are reported to be not able to control hyperlipidemia [22]. Due to the several limitations associated with the use of existing synthetic antidiabetic drugs, the search for newer antidiabetic drugs from natural source continues [23].

## 4. Medicinal Plants as an Alternative Source of Antidiabetic Agents

Natural products, particularly of plant origin, are the main quarry for discovering promising lead candidates and play an imperative role in the upcoming drug development programs [24,25,26]. Ease of availability, low cost, and least side effects make plant-based preparations the main key player of all available therapies, especially in rural areas [27]. Moreover, many plants provide a rich source of bioactive chemicals, which are free from undesirable side effects and possess powerful pharmacological actions [28,29,30,31,32,33,34]. Plants also have always been an exemplary source of drugs with many of the currently available drugs being obtained directly or indirectly from them [2,29,30,31]. The recent review of Durazzo et al. [35] gives a current snapshot of the strict interaction between the main biologically active compounds in plants and botanicals by giving a mini overview of botanicals features, a definition of the study, and examples of innovative (i.e., an assessment of the interaction of bioactive compounds, chemometrics, and the new goal of biorefineries) and a description of existing databases (i.e., plant metabolic pathways, food composition, bioactive compounds, dietary supplements, and dietary markers); in this regard, the authors marked the need for categorization of botanicals as useful tools for health research [35].

For centuries, many plants have been considered a fundamental source of potent antidiabetic drugs. In developing countries, particularly, medicinal plants are used to treat diabetes to overcome the burden of the cost of conventional medicines to the population [2]. Nowadays, treatments of diseases including diabetes using medicinal plants are recommended [36] because these plants contain various phytoconstituents such as flavonoids, terpenoids, saponins, carotenoids, alkaloids, and glycosides, which may possess antidiabetic activities [37]. Also marked by Durazzo et al. [35], the combined action of biologically active compounds (i.e., polyphenols, carotenoids, lignans, coumarins, glucosinolates, etc.) leads to the potential beneficial properties of each plant matrix, and this can represent the first step for understanding their biological actions and beneficial activities. Generally, the main current approaches of study [38,39] of the interactions of phytochemicals can be classified: (i) model system development of interactions [40,41,42]; (ii) study of extractable and nonextractablecompounds [43,44]; or (iii) characterization of biologically active compound-rich extracts [45,46].

The antihyperglycemic effects resulting from treatment with plants are usually attributed to their ability to improve the performance of pancreatic tissue, which is done by increasing insulin secretions or by reducing the intestinal absorption of glucose [2].

The number of people with diabetes today has been growing and causing increasing concerns in the medical community and the public. Despite the presence of antidiabetic drugs in the pharmaceutical market, the treatment of diabetes with medicinal plants is often successful. Herbal medicines and plant components with insignificant toxicity and no side effects are notable therapeutic options for the treatment of diabetes around the world [47]. Most tests have demonstrated the benefits of medicinal plants containing hypoglycemic properties in diabetes management. Ríos et al. [48] described medicinal plants (i.e., aloe, banaba, bitter melon, caper, cinnamon, cocoa, coffee, fenugreek, garlic, guava, gymnema, nettle, sage, soybean, green and black tea, turmeric, walnut, and yerba mate) used for treating diabetes and its comorbidities and the mechanisms of natural products as antidiabetic agents, with attention to compounds of high interest such as fukugetin, palmatine, berberine, honokiol, amorfrutins, trigonelline, gymnemic acids, gurmarin, and phlorizin. The current review of Bindu and Narendhirakannan [49] has categorized and described from literature 81 plants native to Asian countries with antidiabetic, antihyperglycemic, hypoglycemic, anti-lipidemic, and insulin-mimetic properties.

Traditional knowledge of antidiabetic Asian plants: (1) Review in Iran [50,51,52,53,54]; (2) Review in Jordan [55,56,57]; (3)Review in Malaysia [58,59]; (4) Review in Mongolia [60]; (5) Review in Philippines [61,62]; (6) Review in Saudi Arabia [63,64,65]; (7) Review in Korea [66,67,68]; (8) Review in Sri Lanka [69]; (9) Review in Syria [70]; (10) Review in Thailand [71,72,73,74,75]; (11) Review in Turkey [76,77,78,79,80,81,82]; (12) Review in Vietnam [83,84,85]; (13) Review in India [86,87,88,89,90,91,92,93,94,95,96,97,98,99,100,101,102,103,104,105]; and (14) Review in China [99,106,107,108,109,110,111,112].

The biological activities considered in this review are antidiabetic, antihyperglycemic, and hypoglycemic activities as well as α-amylase and α-glucosidase inhibition. A majority of the plant species was tested for antidiabetic activity. The methodology followed while collecting the plant species should influence the treatment of diabetes. Accordingly, the plants screened from the Asian region were selected. Then, the genus name was searched to identify whether any species belonging to the same genus are reported elsewhere. Such plants are listed in Table 1. Those plants where only one species is available are reported in Table 2.

Table 1 has 509 plants belonging to 140 genera. Among these 140 genera, some of them have more than ten species exhibiting an antidiabetic property. *Ficus* with 18 species, *Artemisia* with 13, *Solanum* with 12, *Terminalia* with 11, and *Euphorbia* with 10 are some of the genera which have a large number of species exhibiting an antidiabetic property. In the *Ficus* genus, among 18 plants, the prominent species having relevance to traditional medicines are *Ficus benghalensis*, *Ficus hispida*, and *Ficus elastica*. *Ficus benghalensis*, also known as Indian Banyan tree, is one of the most frequently used plants for the treatment of diabetes [89] and is used in folk medicines, Ayurveda, Unani, Siddha [113], and homeopathy [114]. It is worth mentioning the recent review of Deepa et al. [115] on the role of *Ficus* species in the management of diabetes mellitus: *F. benghalensis*, *F. carica*, *F. glomerata*, *F. glumosa*, *F. racemosa*, and *F. religiosa* exhibited remarkable antidiabetic properties with various mechanisms of action. The leaves and edible fruits of *Ficus hispida* are used for the treatment of diabetes [116] and is used in Ayurveda [117], Siddha [118], and traditional African medicine [119]. *Ficus elastica*, an ethnomedicinal Filipino plant, exhibits less toxicity [62], which is used in diabetes treatment.

In the *Artemisia* genus, *Artemisia absinthium* is one of the traditional medicinal plant used for diabetes treatment [120]. *Artemisia afra* is one of the popular herbal medicines used in the southern part of Africa [121]. *Artemisia herba-alba* is a traditional medicinal plant [122], and its aqueous extract of the leaves and barks reduces blood glucose levels [123]. *Solanum americanum* is a traditional medicine used in Guatemala [124], while *Solanum viarum* is used in India [125]. *Terminalia arjuna* is a plant used in India and Bangladesh [126] and exhibits amylase inhibition (IC_50_ value of 302 μg/mL) [127]. *Terminalia chebula* is a medicinal plant used in India [128], Bangladesh [129], Thailand [75], and Iran [130]. *Euphorbia ligularia* [104], *Euphorbia neriifolia* [131], and *Euphorbia caducifolia* [132] are some of the plants traditionally used in India. Similarly, *Euphorbia thymifolia* and *Euphorbia hirta* are used in Bangladesh [116,133], and *Euphorbia kansui* is a Korean traditional medicinal plant used for diabetes treatment [134]. *Allium cepa*, *Mangifera indica*, *Murraya koenigii*, and *Phyllanthus amarus* reduce triglycerides (TG), total cholesterol (TC), and very low-density lipoproteins (VLDL) levels and exhibit antidiabetic and hypolipidemic effects [135].

α-Amylase inhibitors are reported in several plants, as follows. The corresponding IC_50_ values in μg/mL are in parentheses.

- *Pterocarpus marsupium* (0.9) [136]
- *Catharanthus roseus*, *Carthamus tinctorius*, *Momordica charantia*, *Gynostemma pentaphyllum*, *Glycyrrhiza glabra*, *Smilax glabra*, *Psidium guajava*, and *Rehmannia glutinosa* (ranging from 2.5 to 48.8) [85]
- *Santalum spicatum* (5.43) [136]
- *Ocimum tenuiflorum* (8.9) [128]
- *Rhizoma fagopyri*, *Rosa rugosa*, *Caulis polygoni*, *Fructus amomi*, *Rhizoma alpiniae officinarum*, *Folium ginkgo*, and *Cortex cinnamomi* (16 to 2342.2) [109]
- Methanol extract of *Marrubium radiatum* (61.1) [137]
- *Aloe vera* (80) [138]
- Methanol extract of *Salvia acetabulosa* (91.2) [137]
- *Paronychia argentea* (200) [138]
- Methanol extracts of *Terminalia arjuna* (302) [127]
- Methanol extracts of *Aegle marmelos* (503) [127]
- *Linum usitatisumum* (540) [128]
- Methanol extracts of *Eugenia cumini* (632) [127]
- *Morus alba* (1440) [128]
- *Moringa stenopetala* (1470) [139]
- *Nelumbo nucifera* (2200) [140]
- Aqueous extract of *Costus pictus* (9900) [141]

Alpha-glucosidase inhibitors are reported in several plants, as follows. The corresponding IC_50_ values in μg/mL are in parentheses.

- *Beyeria leshnaultii* (0.39) [136]
- *Mucuna pruriens* (0.8) [136]
- *Acacia ligulata* (1.01) [136]
- *Pterocarpus marsupium* (1.01) [136]
- *Boerhaavia diffusa* (1.72) [136]
- Hydroalcoholic extract of *Juniperus oxycedrus* (4.4) [142]
- *Fagonia cretica* (4.62) [143]
- *Santalum spicatum* (5.16) [136]
- *Rhizoma fagopyri*, *Rosa rugosa*, *Caulis polygoni*, *Fructus amomi*, *Rhizoma alpiniae officinarum*, *Folium ginkgo*, and *Cortex cinnamomi* (49 to 3385.5) [109]
- Methanol extract of *Marrubium radiatum* (68.8) [137]
- Methanol–water extract of *Eugenia polyantha* (71) [144]
- Methanol extract of *Salvia acetabulosa* (76.9) [137]
- Hydroalcoholic extracts of *Ludwigia octovalvis* (202) [145]
- Hydroalcoholic extracts of *Camellia sinensis* (299) [145]
- *Aralia elata* (450) [146]
- Hydroalcoholic extracts of *Iostephane heterophylla* (509) [145]
- *Cinnamomum zeylanicum* (670) [147]
- *Nelumbo nucifera* (1860) [140]
- Aqueous extract of *Costus pictus* (2510) [141]

Table 2 has 194 plant species, which includes only the genera represented by one species.

## 5. Medicinal Plants with Antidiabetic Potential

### 5.1. Preclinical In Vitro/In Vivo (Animal) Studies

Several plant species having hypoglycemic activity have been available in the literature; most of these plants contain bioactive compounds such glycosides, alkaloids, terpenoids, flavonoids, carotenoids, etc., that are frequently implicated as having an antidiabetic effect. In this section, plant species with antidiabetic potential will be organized in alphabetical order (Table 3).

#### 5.1.1. *Acacia arabica* (Fabaceae)

Two doses of chloroform extracts of *Acacia arabica* (250 and 500 mg/kg, p.o. (orally) for two weeks) were evaluated in alloxan-induced diabetic albino rats [891]. The results of this study showed an antidiabetic effect in the two doses tested, decreasing serum glucose level and restoring TC, TG, and high-density lipoprotein (HDL) and low-density lipoprotein (LDL) levels. Additionally, in this study chloroform extracts of *Benincasa hispida* fruit, *Tinispora cordifolia* stem, *Ocimum sanctum* aerial parts, and *Jatropha curcus* leaves were evaluated, showing similar effects.

In another study performed in streptozotocin-induced diabetic rats, the extract of *Acacia arabica* (100 and 200 mg/kg, p.o. for 21 days) provoked a significantly decrease in serum glucose, TC, TG, LDL, and malonyldialdehyde (MDA) levels and a significantly increase in HDL and coenzyme Q10 in a dose-dependent manner [892].

#### 5.1.2. *Achyranthes rubrofusca* (Amaranthaceae)

Hypoglycemic activity of the aqueous and ethanolic extracts of *Achyranthes rubrofusca* leaves was studied in alloxan-induced diabetic rats [893]. The two extracts (200 mg/kg, p.o. for 28 days) significantly decreased the blood glucose level and increased pancreatic enzymes such as superoxide dismutase (SOD), catalase (CAT), and glutathione levels. Better results were obtained with the aqueous extract but were not statistically significant.

#### 5.1.3. *Albizzia lebbeck* (Fabaceae)

Oral administration of a methanol/dichloromethane extract from *Albizzia lebbeck* Benth. stem bark (100, 200, 300, or 400 mg/k, for 30 days) was evaluated in streptozotocin-induced diabetic rats [894]. The treatment significantly decreased fasting blood glucose (FBG) and glycated hemoglobin and enhanced plasma insulin levels. Moreover, it significantly decreased the levels of TC, TG, LDL, and VLDL and significantly increased the level of HDL. The treatment also resulted in a marked increase in reduced glutathione, glutathione peroxidase, CAT, and SOD and a diminished level of lipid peroxidation in liver and kidneys of streptozotocin-induced diabetic rats. Moreover, the histopathological analysis of the pancreas, liver, kidney, and heart showed that the treatment protected these organs in diabetic rats and reduced the lesions in a dose-dependent manner. In another study in streptozotocin-nicotinamide-induced diabetic rats, the methanolic extract of *Albizzia lebbeck* bark significantly decreased the level of serum glucose, creatinine, urea, TC, TG, LDL, and VLDL and increased HDL level [895].

#### 5.1.4. *Aloe vera* (Asphodelaceae)

*Aloe vera* extract was evaluated in streptozotocin-induced diabetic mice and in mouse embryonic NIH/3T3 cells [896]. Administration of an extract at a dosage of 130 mg/kg per day for four weeks resulted in a significant decrease in blood glucose, TG, LDL, and TC, an effect comparable to that of metformin. Moreover, this study showed that a lyophilized aqueous aloe extract (1 mg/mL) upregulated GLUT-4 mRNA synthesis in NIH/3T3 cells. In a more recent study, *Aloe vera* extract (300 mg/kg) exerted antidiabetic effects by improving insulin secretion and pancreatic β-cell function by restoring pancreatic islet mass in streptozotocin-induced diabetic rats [897].

#### 5.1.5. *Amaranthus tricolor* (Amaranthaceae)

Methanolic extract of *Amaranthus tricolor* whole plant at different doses (50, 100, 200, or 400 mg/kg) was administered one hour before glucose administration in the oral glucose tolerance test (GTT) [898]. The results of this study showed significant antihyperglycemic activity in glucose-loaded mice at all doses of the extract tested, with the maximum effect observed at the maximum dose tested and with an effect comparable to glibenclamide (10 mg/kg).

#### 5.1.6. *Anacardium occidentale* (Anacardiaceae)

Hypoglycemic role of *Anacardium occidentale* was reported in streptozotocin-induced diabetic rats [899]. The rats were treated with 175 mg/kg of the aqueous extract, twice daily, beginning 2 days before streptozotocin injection. Three days after streptozotocin administration, there was a significantly lower blood glucose level in pretreated rats compared to control diabetic rats. Moreover, the treatment prevented glycosuria, body weight loss, polyphagia, and polydipsia. A more recent study performed with 100 mg/kg of methanol extract for 30 days showed a decrease of blood glucose levels of streptozotocin-induced diabetic rats and comparable effects to the standard drug Pioglitazone [900].

#### 5.1.7. *Azadirachta indica* (Meliaceae)

One study was designed to evaluate the hypoglycemic effects of different plant extracts (*Azadirachta indica* leaves, *Momordica charantia* fruits, and *Syzygium jambolana* seeds) in single and in combined formulation in alloxan-induced diabetic rabbits [901]. Treatment of diabetes with plant extracts started at 8 days after alloxan injection. A dose of 200 mg/kg of an ethanol extract from the leaves of *Azadirachtaindica* caused a hypoglycemic effect 72 h after administration in diabetic rabbits, with a persistence of up to 24 h.

#### 5.1.8. *Barleria prionitis* (Acanthaceae)

Antidiabetic activity of alcoholic extracts of leaf and root of *Barleria prionitis* (200 mg/kg, p.o. for 14 days) was tested in alloxan-induced diabetic rats [902]. Animals treated with leaf extract significantly decreased blood glucose and glycosylated hemoglobin levels. Moreover, serum insulin and liver glycogen levels were significantly increased. The root extract showed a moderate but nonsignificant antidiabetic activity.

#### 5.1.9. *Bauhinia thoningii* (Fabaceae)

A study conducted on alloxan-induced diabetic rats showed the antidiabetic effect of aqueous leaf extract from *Bauhinia thoningii* [903]. The extract administered orally at a dose of 500 mg/kg for seven days provoked a significant reduction in blood glucose, LDL, and coronary risk index.

#### 5.1.10. *Caesalpinia ferrea* (Fabaceae)

Aqueous extract of the stem bark of *Caesalpinia ferrea* (300 and 450 mg/kg, daily for four weeks) was administered orally to streptozotocin-induced diabetic rats [904]. The results of this study showed a significant reduction of blood glucose levels and an improvement of the metabolic state of the animals (low levels of TC, TG, and epididymis adipose tissue).

#### 5.1.11. *Camellia sinensis* (Theaceae)

The hypoglycemic activity of the crude tea leaves extract of *Camellia sinensis* was investigated on streptozotocin-induced diabetic mice [905]. The tea (0.5 mL/day) was administered for 15 and 30 days and caused antihyperglycemic and hypolipidemic (TG and TC) activities in diabetic rats. Moreover, protective effects such as recovery of certain altered hematobiochemical parameters—creatinine, urea, uric acid, aspartate aminotransferase (AST), and alanine aminotransferase (ALT)—and reduced body weight were observed.

#### 5.1.12. *Casearia esculenta* (Flacourtiaceae)

The extract of *Casearia esculenta* root in streptozotocin-induced diabetic rats (200 and 300 mg/kg, p.o. for 45 days) significantly restored levels of glucose, urea, uric acid, creatinine, and albumin; the albumin/globulin ratio; and the activities of diagnostic marker enzymes AST, ALT, alkaline phosphatase (ALP), and γ-glutamyltranspeptidase (GGT) [906].

#### 5.1.13. *Cassia fistula* (Fabaceae)

Alcoholic extracts of stem bark of *Cassia fistula* administered to alloxan-induced diabetic rats at 250 or 500 mg/kg for 21 days significantly decreased blood glucose levels [907]. The extract also recovered normal levels of serum cholesterol, TG, creatinine, albumin, total proteins, and body weight. Moreover, the alcoholic extract showed significant antioxidant activity by reducing 2,2-diphenyl-1-picrylhydrazyl (DPPH), nitric oxide, and hydroxyl radical induced in vitro.

#### 5.1.14. *Cassia grandis* (Fabaceae)

The aqueous and ethanolic extracts of *Cassia grandis* (150 mg/kg, p.o. for 10 days treatment) were evaluated for antidiabetic activity by a GTT in normal rats and alloxan-induced diabetic rats [908]. The two extracts showed antidiabetic potential, decreasing the blood glucose, TC, and TG levels.

#### 5.1.15. *Catharanthus roseus* (Apocynaceae)

Dichloromethane-methanol extracts of *Catharanthus roseus* leaves and twigs in streptozotocin-induced diabetic rats significantly reduced blood glucose levels and hepatic enzyme activities of glycogen synthase, glucose 6-phosphate-dehydrogenase, succinate dehydrogenase, and malate dehydrogenase [909]. In another study performed in streptozotocin-induced diabetic rats, the ethanolic extracts of *Catharanthus roseus* (100 and 200 mg/kg) detrained the glucose transport system in the liver for 4 weeks and significantly amplified the expression of the GLUT gene [711].

#### 5.1.16. *Cecropia pachystachya* (Urticaceae)

The hypoglycemic effect of the methanolic extract from the leaves of *Cecropia pachystachya* was tested in normal, glucose loading, and alloxan-induced diabetic rats [910]. The methanolic extract provoked a significant hypoglycemic effect, which resulted in a 68% reduction of blood glucose after 12 h of induction. Moreover, the extract presented relevant antioxidant activity with IC_50_ = 3.1 µg/mL (DPPH assay) and EC_50_ = 10.8 µg/mL (reduction power).

#### 5.1.17. *Ceriops decandra* (Rhizophoraceae)

The antidiabetic effects of daily oral administration of an ethanolic extract from *Ceriops decandra* leaves (30, 60, and 120 mg/kg) for 30 days were evaluated in normal and alloxan-induced diabetic rats [911]. Oral administration of 120 mg/kg of the extract modulated all the determined parameters (blood glucose, hemoglobin, liver glycogen, and some carbohydrate metabolic enzymes) to levels seen in control rats. Furthermore, these dose effects were comparable to those of glibenclamide.

#### 5.1.18. *Chiliadenus iphionoides* (Asteraceae)

The ethanolic extracts of *Chiliadenus iphionoides* aerial parts increased insulin secretion from β cells and glucose uptake by adipocytes and skeletal myotubes, in vitro [912]. Moreover, a 30-day oral starch tolerance test was performed on a sand rat, showing hypoglycemic activity.

#### 5.1.19. *Cinnamomum cassia* and *Cinnamomum japonica* (Lauraceae)

Cinnamon bark extracts were administered at doses of 200 and 300 mg/kg for 14 days in high-fat, diet-fed, and low-dose streptozotocin-induced diabetic mice [913]. The results of this study showed that *Cinnamomum cassia* and *Cinnamomum japonica* bark extracts significantly decreased blood glucose concentration. Also, cinnamon extracts significantly increased the consumption of extracellular glucose in insulin-resistant HepG2 cells and normal HepG2 cells compared with controls, suggesting an insulin sensitivity improvement.

#### 5.1.20. *Citrullus colocynthis* (Cucurbitaceae)

The effect of root extracts of *Citrullus colocynthis* was investigated on the biochemical parameters of normal and alloxan-induced diabetic rats [914]. Aqueous extracts of the roots showed a significant reduction in blood sugar levels when compared with chloroform and ethanol extracts. Moreover, the aqueous extract improved body weight and serum creatinine, urea, protein, and lipids and restored levels of total bilirubin, conjugated bilirubin, AST, ALT, and ALP. In another study in alloxan-induced diabetic rats, *Citrullus colocynthis* aqueous seed extract stabilized animal body weight and ameliorated hyperglycemia in a dose- and time-dependent manner, which was attributable to the regenerative effect on β cells and intra-islet vasculature [915].

#### 5.1.21. *Coscinium fenestratum* (Menispermaceae)

Alcoholic extract of the stems of *Coscinium fenestratum* in streptozotocin-nicotinamide-induced diabetic rats regulates glucose homeostasis and decreased gluconeogenesis [916]. The drug also has a protective action on cellular antioxidant defense.

#### 5.1.22. *Eucalyptus citriodora* (Myrtaceae)

Aqueous extract of *Eucalyptus citriodora* leaf in alloxan-induced diabetic rats (250 and 500 mg/kg, p.o. for 21 days) significantly reduced blood glucose levels [917].

#### 5.1.23. *Gymnema sylvestre* (Apocynaceae)

An ethanolic extract of *Gymnema sylvestre* leaf (100 mg/kg, p.o. for 4 weeks) was examined in vitro and in vivo to investigate the role of antioxidants in streptozotocin-induced diabetic rats [918]. The ethanol extract showed antihyperglycemic activity and improved the antioxidant status in diabetic rats. Moreover, the extract showed in vitro antioxidant activity in thiobarbituric acid (TBA), SOD, and 2,2-azino-bis-3-ethylbenzthiazoline-6-sulphonic acid assays.

#### 5.1.24. *Heinsia crinata* (Rubiaceae)

Ethanolic extract of *Heinsia crinata* leaf in alloxan-induced diabetic rats (450–1350 mg/kg, p.o. for two weeks) significantly reduced the FBG levels [919].

#### 5.1.25. *Helicteres isora* (Sterculiaceae)

Butanol and aqueous ethanol extracts of *Helicteres isora* root (250 mg/kg, p.o. for 10 days) were investigated in alloxan-induced diabetic rats [920]. The two treatments reduced blood glucose, TC, TG, and urea levels. Further histological examination showed the restoration of pancreatic islets, kidney glomeruli, and liver to their normal sizes.

#### 5.1.26. *Momordica charantia* (Cucurbitaceae)

One study evaluated the antihyperglycemic and antioxidative potential of aqueous extracts of *Momordic charantia* pulp and *Trigonella foenum-graecum* seed in alloxan-induced diabetic rats [921]. The *Momordica charantia* extract treatment for 30 days significantly decreased the blood glucose levels and showed antioxidant potential to protect vital organs such as heart and kidney against damage caused by diabetes-induced oxidative stress. Furthermore, a similar activity was found with the *Trigonella foenum-graecum* extract treatment. In another study already reported [901], an antidiabetic effect from *Momordica charantia* leaves (200 mg/kg) was observed in rabbits 72 h after they were fed a methanolic extract. In a recent study performed in streptozotocin-induced diabetic rat, the treatment of 400 mg/kg of ethanol extract significantly decreased body weight, serum glucose, insulin TNF-α, and interleukin 6 (IL-6) [922].

#### 5.1.27. *Moringa oleifera* (Moringaceae)

One study investigated the antidiabetic and antioxidant effects of methanol extracts of *Moringa oleifera* pods (150 and 300 mg/kg, p.o. for 21 days) in streptozotocin-induced diabetic rats [923]. Both doses induced a significant reduction in serum glucose and nitric oxide levels, with a concomitant increase in serum insulin and protein levels. Furthermore, the methanol extracts increased antioxidant levels in pancreatic tissue and concomitantly decreased TBA levels. Additionally, a histological pancreas examination showed that *Moringa oleifera* treatment significantly reversed the histoarchitectural damage to islet cells provoked by induced diabetes. In a recent study performed in alloxan-induced diabetic rats, the consumption of the *Moringa oleifera* leaves showed a hypoglycemic effect and prevented body weight loss [924].

#### 5.1.28. *Murraya koenigii* (Rutaceae)

Aqueous extract of *Murraya koenigii* leaf in alloxan-induced diabetic rats (200, 300, and 400 mg/kg) significantly reduced blood glucose level and was found to have a beneficial effect on carbohydrate metabolism [458]. Moreover, the ethanolic extract of this plant, in mice, ameliorates dexamethasone-induced hyperglycemia and insulin resistance in part by increasing glucose disposal into skeletal muscle [925].

#### 5.1.29. *Opuntia ficus-indica* (Cactaceae)

Various extracts from edible *Opuntia ficus-indica* (petroleum ether, ethyl acetate, butanolic, aqueous, and water parts) and a standard drug as a positive control (dimethyl biguanide, 100 mg/kg) were tested in streptozotocin-induced diabetic mice [926]. The results of this study showed that all extracts tested significantly decreased blood glucose levels and maintained body weight, except the aqueous extract. Mainly, the petroleum ether extract showed a remarkable decrease in blood glucose levels.

#### 5.1.30. *Origanum vulgare* (Lamiaceae)

The phytochemical analysis of methanolic extract from *Origanum vulgare* showed an enriched composition in biophenols, and it has demonstrated in vitro antioxidant activity in DPPH assays [927]. An in vivo study performed in streptozotocin-induced diabetic mice with methanolic and aqueous extract showed that aqueous extract had no impact on diabetes induction, while methanolic extract reduced diabetes incidence and preserved normal insulin secretion. Moreover, methanolic extract upregulated antioxidant enzymes (SOD, CAT, glutathione reductase, and peroxidase), attenuated pro-inflammatory activity, and showed cytoprotective activity.

#### 5.1.31. *Passiflora nitida* (Passifloraceae)

Hydroethanolic leaf extract from *Passiflora nitida* showed an α-glucosidase IC_50_ = 6.78 ± 0.31 μg/mL and α-amylase IC_50_ = 93.36 ± 4.37 in vitro [928]. Also, in vivo experiments testing different saccharide tolerances revealed significant glycemic control. Moreover, in alloxan-induced diabetic mice, these assays showed a decrease in TC, a hypoglycemic effect, and antioxidant activity based on the measurement of TBA.

#### 5.1.32. *Paspalum scrobiculatum* (Poaceae)

Antidiabetic activity of aqueous and ethanolic extracts of grains of *Paspalum scrobiculatum* Linn. was evaluated in alloxan-induced diabetic rats [929]. The extracts at 250 and 500 mg/kg, p.o. for 15 days treatment, significantly reduced the blood glucose level and lipid parameters in a dose-related manner. Also, the extract treatment showed a significant increase in the liver glycogen and a significant decrease in glycated hemoglobin levels.

#### 5.1.33. *Persea americana* (Lauraceae)

The hydroalcoholic extract of the leaves of *Persea americana* (0.15 and 0.3 g/kg, p.o. daily for 4 weeks) reduced blood glucose levels in streptozotocin-induced diabetic rats [930]. The extract did not affect the plasma insulin level, suggesting that the hypoglycemic effect was due to extrapancreatic activity, independent of insulin secretion. Additionally, the extract improved the metabolic state of diabetic animals and increased body weight. In another study, the aqueous extract of *Persea americana* seeds significantly decreased glucose levels and reversed the histopathological damage that occurred in alloxan-induced diabetic rats, comparable to the effects of glibenclamide [931].

#### 5.1.34. *Phoenix dactylifera* (Arecaceae)

Antidiabetic effects of leaf extract of *Phoenix dactylifera* at 100, 200, and 400 mg/kg, p.o. and its fractions at 50, 100, and 200 mg/kg, p.o. for 14 days treatment were evaluated in alloxan-induced diabetic rats [932]. The treatment showed a significant reduction of blood glucose, TC, and TG levels and water intake and a significant increase of plasma insulin levels compared to the control group.

#### 5.1.35. *Phyllanthus niruri* (Euphorbiaceae)

The methanol extract of aerial parts of *Phyllanthus niruri* was evaluated in alloxan-induced diabetic rats [933]. The results of this study showed a significant reduction of blood glucose, TC, and TG levels in a dose-related manner. Moreover, histological analyses showed that that extract had imparted cell regenerative power. In another study was observed that a *Phyllanthus niruri* leaf aqueous extract improves kidney functions; ameliorates kidney oxidative stress, inflammation, fibrosis, and apoptosis; and enhances kidney cell proliferation in adult male rats with diabetes [934].

#### 5.1.36. *Phyllanthus simplex* (Euphorbiaceae)

The hypoglycemic effect of *Phyllanthus simplex* fractions was evaluated in normal and alloxan-diabetic diabetic rats [935]. Petroleum ether (200 and 400 mg/kg), ethyl acetate (100 and 200 mg/kg), methanol (125 and 250 mg/kg), and water fraction (150 and 300 mg/kg) were investigated for 21 days. Methanol and water fractions showed a significant antihyperglycemic effect and restored the antioxidant enzyme levels in liver and kidney.

#### 5.1.37. *Picralima nitida* (Magnoliopsida)

The antidiabetic activity of *Picralima nitida* was tested in streptozotocin-induced diabetic mice [936]. In vitro examination of a hydroethanolic extract from the whole plant showed antioxidant activity using DPPH and showed an IC_50_ = 0.24 mg/mL. The extract (300 mg/kg) revealed significant hypoglycemic activity. Also, the measurement of stress markers in plasma, liver, and kidneys showed high antioxidant potential.

#### 5.1.38. *Piper longum* (Piperaceae)

In a study with an aqueous extract from *Piper longum* root was administered a dose of 200 mg/kg in male albino rats, with diabetes induced by intraperitoneal administration of streptozotocin; these rats presented significant antidiabetic activity after 6 h of treatment, with better effectiveness than glibenclamide [937]. Administration of the aqueous extract at the same dose for 30 days in streptozotocin-induced diabetic rats resulted in a significant reduction in blood glucose levels and correction of diabetic dyslipidemia compared with untreated diabetic rats. There was a significant reduction in the activities of liver and renal function markers in treated diabetic rats compared with untreated diabetic rats, indicating that the extract has a protective effect against liver and kidney damage and that it is nontoxic. Therefore, the plant extract is capable of managing hyperglycemia and complications of diabetes in streptozotocin-induced diabetic rats.

#### 5.1.39. *Sonchus oleraceus* (Asteraceae)

The antidiabetic activity of *Sonchus oleraceus* was tested in streptozotocin-induced diabetic mice [936]. In vitro examination of a hydroethanolic extract from the whole plant showed antioxidant activity using DPPH and showed an IC_50_ = 0.19 mg/mL. The extract showed significant antidiabetic activity, and measurement of stress markers in plasma, liver, and kidneys showed high antioxidant potential. The effects may be attributed to the significant free radical-scavenging capacity, hypoglycemic activity, and the ability to prevent oxidative stress in diabetic rats, which was determined by the decrease of MDA and H_2_O_2_ and the increase in CAT activity.

#### 5.1.40. *Syzygium jambolana* (Myrtaceae)

As we have commented, a combination of *Syzygium jambolana* extract obtained from the seeds, fruits of *Momordica charantia*, and leaves of *Azadirachta indica* (200 mg/kg) showed a hypoglycemic effect in rabbits [901]. Treatment of diabetes with plant extracts was started at 8 days after alloxan injection. The antidiabetic effect was produced after 72 h in many of the rabbit’s groups. This effect may be due to enhanced endogenous insulin production, possibly through pancreatic β-cell regeneration or repair caused by higher insulin levels in the serum.

#### 5.1.41. *Tamarindus indica* (Fabaceae)

In vitro assays of an alcoholic extract made from *Tamarindus indica* stem bark showed significant antioxidant activity in DPPH, nitric oxide, and hydroxyl radical [907]. Alloxan-induced diabetic rats were treated orally with the alcoholic extract from *Tamarindus indica* at 250 and 500 mg/kg doses for 21 days, and a significant decrease of blood glucose levels was observed. In another study, hydroethanolic seed coat extract of *Tamarindus indica* significantly reduced blood glucose levels in normoglycaemic, glucose loaded, and alloxan-induced diabetic rats [938].

#### 5.1.42. *Terminalia chebula* (Combretaceae)

Chloroform extract of *Terminalia chebula* seed powder in streptozotocin-induced diabetic rats (100, 200, and 300 mg/kg) significantly reduced the blood glucose level in a dose-dependent manner and presented a potent renoprotective action [939].

#### 5.1.43. *Terminalia catappa* (Combretaceae)

The antidiabetic potential of petroleum ether, methanol, and aqueous extract of *Terminalia catappa* fruits in alloxan-induced diabetic rats was performed [940]. All three extracts reduced FBG levels.

#### 5.1.44. *Trigonella foenum-graecum* (Fabaceae)

The antidiabetic effects of ethanol extract of *Trigonella foenum-graecum* seeds in alloxan-induced diabetic rats at different doses (0.1, 0.5, 1, and 2 g/kg) were evidenced, showing significant blood glucose-lowering capacity [941]. Moreover, the hydroalcohol extract of *Trigonella foenum-graecum* seed attenuates markers of inflammation and oxidative stress while improving exocrine function in alloxan-induced diabetic rats [942].

#### 5.1.45. *Vaccinium arctostaphylos* (Ericaceae)

The effects of ethanolic extract of *Vaccinium arctostaphylos* fruit was investigated in alloxan-diabetic rats for three weeks [943]. The treatment significantly decreased the blood glucose and TG levels and increased the erythrocyte SOD, glutathione peroxidase, CAT activities, and expression of GLUT-4 and insulin genes.

#### 5.1.46. *Vernonia amygdalina* (Asteraceae)

One study investigated the antidiabetic activity of the various combinations of metformin (50 mg/kg) and aqueous extracts of *Vernonia amygdalina* leaves (100 mg/kg) in normoglycemic and alloxan-induced diabetic rats [944]. Results showed that the combinations of the extract and metformin caused more reduction in glycemia compared to any of the agents acting alone in either of the two categories of animals.

#### 5.1.47. *Witheringia solanacea* (Solanaceae)

Normal rats were treated with an aqueous extract from *Witheringia solanacea* leaves at 250, 500, and 1000 mg/kg doses, and only the last two doses significantly decreased blood glucose levels after 1 h of a GTT [945]. Moreover, the 500 mg/kg dose significantly reduced blood glucose levels in alloxan-induced hyperglycemic rats at 4 h and 5 h of treatment.

#### 5.1.48. *Zaleya decandra* (Aizoaceae)

Oral administration of an ethanolic extract from *Zaleya decandra* roots (200 mg/kg, for 15 days) significantly restored the levels of glucose, TC, TG, total proteins, urea, creatinine, lipid peroxidation, and antioxidant enzymes in alloxan-induced diabetic rats [946]. Moreover, histopathological analysis showed significant regenerative power in the extract-treated group compared to the control group, including effects in necrosis and degeneration in the liver and pancreas.

#### 5.1.49. *Zizyphus mauritiana* (Rhamnaceae)

Petroleum ether and aqueous extract of *Zizyphus mauritiana* (200 and 400 mg/kg, p.o. for seven days) in alloxan-induced diabetic rats significantly restored elevated biochemical parameters such as glucose, urea, creatinine, TC, TG, HDL, LDL, hemoglobin, and glycosylated hemoglobin [947].

## 6. Phytochemicals with Antidiabetic Potential

Discovery of the new natural antidiabetic drugs could be great promise due to minimal efficacy and safety concerns of current antidiabetic drugs for the hundreds of millions of individuals which are currently seeking better management of diabetes [948]. In this relation, the investigation of phytochemicals responsible for antidiabetic effects has progressed in the last few decades. The antidiabetic effect of plant materials have been attributed to the mixture of phytochemicals or a single component of plant extracts. Medicinal plants produce a wide variety of phytochemicals, include alkaloids, phenolic acids, flavonoids, glycosides, saponins, polysaccharides, stilbenes, and tannin, which are intensively investigated for their antidiabetic effects. In Table 4 are represented sources, structures, and targets of some potential antidiabetic phytochemicals. The beneficial effect of phytochemicals can be through various mechanisms such as regulation of glucose and lipid metabolism, insulin secretion, stimulating β cells, NF-*k*B signalling pathway, inhibition of gluconeogenic enzymes, and reactive oxygen species (ROS) protective action.

### 6.1. Alkaloids

The following alkaloids—berberine, boldine, lupanine neferin, oxymatrine, piperine, and sanguinarine—are studied for their antidiabetic activity. Christodoulou et al. [949] discussed the antidiabetic impact of certain alkaloids, with special reference to their molecular targets throughout the insulin-signaling pathway: in vitro and in vivo evidence support the effects of berberine, trigonelline, piperine, oxymatrine, vindoneline, evodiamine, and neferine on insulin-signaling and related cascades in β cells, myocytes, adipocytes, hepatocytes, and other cells; the authors concluded that in-depth molecular studies are needed as well as large clinical trials to assess their potential as antidiabetic agents [949].

Berberine is an isoquinoline alkaloid, isolated from medicinal plants of *Berberis* (Berberidaceae). It has an antihyperglycaemic activity by decreasing absorption of glucose [950]. Berberine was reported to inhibit α-glucosidase and to decrease glucose transport through the intestinal epithelium [950,951]. It has a particular interest in the management of T2DM and cardiovascular diseases due to potent antioxidant, anti-inflammatory, glucose-lowering, and lipid-lowering properties [952].

Boldine is a benzylisoquinoline class alkaloid, isolated from *Peumus boldus* Moliba (Chilean boldo tree, family Monimiaceae) [953]. Boldine improves endothelial function in diabetic db/db mice through inhibition of angiotensin II-mediated BMP4 oxidative stress cascade. It reduces overproduction of ROS by inhibiting Ang II-stimulated BMP4 expression [954].

Lupanine is a quinolizidine alkaloid, isolated from *Lupinus* species, particularly from *Lupinus perennis.* It enhances insulin secretion [955]. Recently, Wiedemann et al. [956] showed how lupanine improves glucose homeostasis by influencing ATP-sensitive potassium (KATP) channels and insulin genes.

Another antidiabetic alkaloid molecule is neferine; it is a bisbenzyl isoquinoline alkaloid isolated from the *Nelumbo nucifera* (Nelumbonaceae). It decreased the expression of CCL5 and CCR5 mRNA in the superior cervical ganglion of T2DM rats. After treatment with neferine 4 mg/kg for 4 weeks, body weight, FBG, blood pressure, TC, and TG were reduced and high-density lipoprotein was increased [957].

Oxymatrine is an alkaloid of the class quinolizidine obtained from the root of *Sophora flavescens* (family Fabaceae). It decreased blood glucose, urinary protein and albumin excretion, serum creatinine, and blood urea nitrogen in a T2DM high-fat diet streptozotocin (HFD-STZ) nephropathy model at an oral dose of 150 mg/kg per day for 11 weeks [953,958].

Piperine is a natural alkaloid present in *Piper* species fruits. It has bio-enhancing effects with metformin in lowering blood glucose levels [959].

Sanguinarine is a benzophenanthridine alkaloid; it is an excellent intercalator of DNA and RNA. Sanguinarine was targeted as a candidate agent for T2DM treatment by a computational bioinformatics approach [960].

### 6.2. Flavonoids

Flavonoids represent a large class of plant secondary metabolites found in a wide range of fruits, vegetables, and herbs. Due to the presence of hydroxyl groups and aromatic rings of the flavonoid structures, they can play as natural antioxidants. Flavonoid-containing products are commonly used in antidiabetic diets. Many flavonoids such as catechins, fisetin, kaempferol, luteolin, naringenin, quercetin, rutin, morin, silymarin, chrysin, baicalein, icariin, isoliquiritigenin, diosmin, isoangustone A, genistein, and others were tested for their antidiabetic properties. For instance, the current work of Den Hartogh and Tsiani, [961] summarizes well the in vitro and in vivo animal studies on the antidiabetic effects of naringenin; as shown by authors among the effects reported, naringenin can reduce glucose adsorption by the intestinal brush border, reduce renal glucose reabsorption, and increase glucose uptake and use by muscle and fat tissues; in hepatocytes, naringenin treatment reduces TG production and gluconeogenesis, resulting in the attenuation of hyperglycemia and hyperlipidemia [961]. The authors concluded that naringenin could be seen as a prime candidate for medicinal use against insulin resistance and T2DM and highlighted how more human studies are required in this direction [961].

Catechins (catechin, epicatechin, and epigallocatechin gallate (EGCG)) are the major active components of tea and cacao products. The protective effects against oxidative damage and enhancing SOD, glutathione S-transferase (GST), and CAT activities of catechins are well demonstrated. However, some studies reported that they did not find a hypoglycemic effect of an extract of green and black tea in adults with T2DM [962].

The flavonoid fisetin presents in a wide variety of plants. Fisetin significantly reduces blood glucose, improves glucose homeostasis through the inhibition of gluconeogenic enzymes, and increases the level and activity of glyoxalase 1 [963,964,965].

Kaempferol as a natural flavonol is found in a variety of plants. It acts as an antioxidant by reducing oxidative stress. It promotes insulin sensitivity and preserves pancreatic β-cell mass [966].

Luteolin is a flavone, present in many aromatic flowering plants, including members of the Lamiaceae. It was recommended for treating diabetic nephropathy. Luteolin ameliorates cardiac failure in T1DM cardiomyopathy [967,968].

Naringenin is a naturally occurring flavanone predominantly found in grapefruit [953]. It attenuates diabetic nephropathy via its anti-inflammatory and anti-fibrotic activities [953,969]. Naringenin also decreased expression of interleukin (IL)-1β, IL-6, type IV collagen, fibronectin, and transforming growth factor β1 [969].

Quercetin is a natural flavonol; it is present in the composition of a number biological active additives as well as in food additives. The protective effects of quercetin on diabetes have been intensively investigated. It decreased the cell percentages of G(0)/G(1) phase, Smad 2/3 expression, laminin, and type IV collagen and TGF-β (1) mRNA levels. Quercetin also activated the Akt/cAMP response element-binding protein pathway [970,971].

Rutin is a natural flavonoid glycoside present in many types of fruits and vegetables. It improves glucose homeostasis by altering glycolytic and gluconeogenic enzymes. It is also involved in stimulatory effects on glucose uptake. Rutin enhances insulin-dependent glucose transporter and potentiates insulin receptor kinase [972,973,974].

Another natural flavonoid molecule, morin, is isolated from *Morus alba*, *Maclura pomifera*, *Psidium guajava*, *Chlorophora tinctoria*, *Prunus dulcis*, *Maclura tinctoria*, *Castanea sativa*, and many other plant species. It as an activator and sensitizer of the insulin receptor stimulating the metabolic pathways. It was also found to rescue endothelial dysfunction in a diabetic mouse model by activating the Akt/eNOS pathway [975,976]. Recently, Razavi et al. [977] showed how morin improves diabetic conditions through downregulation of the miR-29a level. Currently, Pandey et al. [978], by exploring the role of Morinin modulating ER stress in STZ/nicotinamide-induced type 2 diabetic male Wistar rats, demonstrated how morin attenuates ER stress throughout the downregulation of the PERK-eIF2α-ATF4 pathway (PERK endoplasmic reticulum kinase; eIF2α eukaryotic initiation factor 2 alpha; ATF4 activating transcription factor 4) by interacting with the PERK protein; the authors concluded that the anti-ER stress and antihyperglycemic potential of Morin opens new possibilities for the exploitation of the use of morin as a bioactive supplement in managing ER stress during type 2 diabetes.

Silymarin is a complex of flavonoids containing silybin, silydianin, and silychrisin isolated from the milk thistle plant [979,980,981]. It has nephroprotective effects in T2DM and can reduce blood glucose levels [982]. Currently, Meng et al. [983] showed that silymarin ameliorates diabetic cardiomyopathy through the inhibition of TGF-β1/Smad signaling, suggesting that silymarin could have a potential role in diabetic cardiomyopathy treatment.

Chrysin [984] is a naturally occurring flavone, predominantly found in *Passiflora caerulea*, *Passiflora incarnata*, and *Oroxylum indicum* [953]. It suppressed transforming growth factor-beta (TGF-β), fibronectin, and collagen-IV protein expressions in renal tissues. Chrysin also reduced the serum levels of pro-inflammatory cytokines, interleukin-1beta (IL-1β), and IL-6 [985]. Taslimi et al. [986] studied the antidiabetic and anticholinergic effects of chrysin on cyclophosphamide-induced multiple organ toxicity in rats by focusing on pharmacological evaluation of some metabolic enzyme activities: chrysin exhibited an ameliorative effect against CYP-induced brain, heart, liver, testis, and kidney toxicity.

Baicalein is a flavonoid found in *Oroxylum indicum*, *Scutellaria baicalensis*, and other species. It mitigates oxidative stress, suppresses the activation of NF-κB, and decreases expression of iNOS and TGF-β1. It also normalizes the levels of serum proinflammatory cytokines and liver function enzymes [953,987].

### 6.3. Terpenoids

#### 6.3.1. Triterpenoids

The review of Hamid et al. [988] highlights recent findings on the chemistry and bioactivities of tetracyclic triterpenoids (i.e., dammarane, cucurbitane, cycloartane, lanostane, and protostane groups) from some plants such as *Panax ginseng*, *Panax quinquefolium*, *Panax notoginseng*, *Gynostemma pentaphyllum*, *Astragalus membranaceus*, *Momordica charantia*, and *Ganoderma lucidum*. Alqahtani et al. [989] summarized the multiple biological activities on glucose absorption; glucose uptake; insulin secretion; diabetic vascular dysfunction; and retinopathy and nephropathy of oleanolic acid, glycyrrhizin, glycyrrhetinic acid, ursolic acid, betulin, betulinic acid and lupeol, examples of pentacyclic triterpenoids.

Boswellic acids are pentacyclic triterpene found in the oleo-gum-resin from the trees of different *Boswellia* species (*Boswellia serrata* and *Boswellia carteri*). The activity has been attributed to stimulating β cells to release more insulin. They are used for the prophylaxis and treatment of damage and inflammation of the islets of langerhans [990,991].

The natural triterpene celastrol is found in *Tripterygium wilfordii*, *Celastrus orbiculatus*, *Celastrus aculeatus*, *Celastrus reglii*, *Celastrus scandens*, and other plant species. Protective effects of celastrol were investigated on diabetic liver injury via TLR4/MyD88/NF-*k*B signaling pathway in T2DM. It suppresses the obesity process via increasing antioxidant capacity and improving lipid metabolism. Celastrol is an NF-*k*B inhibitor, improves insulin resistance, and attenuates renal injury [992,993,994].

Oleanolic acid is a pentacyclic triterpenoid that exists widely in nature in fruits, herbs, and vegetables. Recent reports have highlighted the benefits of oleanolic acid in the prevention and treatment of T2DM [995]. Zeng et al. [996] reported that oleanolic acid reduces hyperglycemia beyond the treatment period with Akt/FoxO1-induced suppression of hepatic gluconeogenesis in T2DM mice.

Another pentacyclic triterpenoid is ursolic acid that can be extracted from berries, leaves, flowers, and fruits of medicinal plants such as *Eriobotrya japonica*, *Calluna vulgaris*, *Rosmarinus officinalis*, and *Eugenia jambolana* [948].

Many studies have shown that ursolic acid can directly inhibit PTP1B and improve insulin sensitivity [997,998]. It improves blood glucose levels in mice characterized by diet-induced obesity [999]. Ling reported that ursolic acid provides kidney protection in diabetic rats [1000].

#### 6.3.2. Diterpenoids

Triptolide is a diterpenoid with three epoxide groups, isolated from *Tripterygium wilfordii*. Triptolide reduced the levels of phosphorylated protein kinase B and phosphorylated inhibitor of kappa B and increased caspases 3, 8, and 9. Triptolide treatment is accompanied by alleviated glomerular hypertrophy and podocyte injury [1001,1002].

#### 6.3.3. Polysaccharides

Galactomannan is a polysaccharide isolated from the tubers of *Amorphophallus konjac* and seeds of *Cyamopsis tetragonolobus.* It can delay the rate of glucose absorption and, thereby, helps to reduce postprandial hyperglycemia [1003,1004].

Another carbohydrate is inulin; *Helianthus tuberosus* tubers contain 75 to 80% inulin. It is a well-known remedy in diabetic treatment. It can act as a biogenetic for the development of natural intestinal microflora after dysbacteriosis and in the modulation of blood metabolites and liver enzymes [1005,1006].

#### 6.3.4. Miscellaneous

Resveratrol improves health and survival of mice on a high-calorie diet [1007]. Piceatannol, a resveratrol derivative, promotes glucose uptake through glucose transporter 4 translocation to the plasma membrane in L6 myocytes and suppresses blood glucose levels in T2DM model db/db mice [1008].

Piceatannol lowers the blood glucose level in diabetic mice [1009]. Intravascular administration of piceatannol enhanced glucose tolerance in freely moving healthy rats [1010]. Resveratrol analog piceatannol restores the palmitic acid-induced impairment of insulin signaling and production of endothelial nitric oxide via activation of anti-inflammatory and antioxidative heme oxygenase-1 in human endothelial cells [1011]. Vallianou et al. [1012] described how the antihyperglycemic effects of resveratrol seem to be the results of increased activity of the glucose transporter in the cytoplasmic membrane; the authors marked that the main antihyperglycemic actions of resveratrol are attributed to the activation of SIRT1 with the involvement of AMPK (5′ AMP-activated protein kinase). Szkudelski and Szkudelska [1013] gave an overview of the role of resveratrol in diabetes from animal models to human studies; in particular, the authors summarized the effect of resveratrol reported in animals models: improvement of glucose homeostasis, decrease of insulin resistance, protection of pancreatic β cells, improvement of insulin secretion, and amelioration of metabolic disorders [1013]. As marked by the same authors [1013], the antidiabetic activity of resveratrol can be related to the resveratrol capability to increase expression/activity of AMPK and SIRT1 in various tissues of diabetic subjects. Bagul and Banerjee described well the multi-target effects against diabetes of resveratrol [1014]. They illustrated the improvement of insulin sensitivity, enhancement of GLUT-4 translocation, reduction of oxidative stress, regulation of carbohydrate metabolizing enzymes, activation of SIRT1 and AMPK, and decrease of adipogenic genes. The current study of Öztürk et al. [1015] provides a critical overview of currently available clinical studies examining the effects of resveratrol in DM in last decade:

Butein is a natural phenolic chalcone, isolated from many plant species, including *Toxicodendron vernicifluum*, *Dalbergia odorifera*, *Cyclopia subternata*, *Semecarpus anacardium*, and *Creopsis tungtoria.* Butein inhibits central NF-*k*B signalling and improves glucose homeostasis [1016].

Curcumin is a natural polyphenol; it has two *o*-methoxy phenolic groups, one enone moiety and an α, β-unsaturated diketone group. It exhibits keto-enol tautomerism [1017].

Kunwar and Priyadarsini reported that curcumin reduces blood glucose and glycosylated hemoglobin levels and prevented weight loss. It was also reported to reduce several other complications associated with diabetes like fatty liver, diabetic neuropathy, diabetic nephropathy, vascular diseases, musculoskeletal diseases, and islet viability [1017,1018,1019].

Tocotrienol and tocopherol are commonly known as vitamin E. They are isomers and are found in a wide variety of plants [1020]. Haghighat et al. [1021] demonstrated that supplementation of tocotrienol at 15 mg daily for 4 weeks caused a significant reduction of the high-sensitivity C-reactive protein in a group of patients with T2DM. Kuhad and Chopra [1022] reported that tocotrienol attenuates diabetic nephropathy by the involvement of the NF-*k*B signaling pathway, oxidative-nitrosative stress, and inflammatory cascade in the experimental model.

Indole-3-carbinol is the nutritive phytochemical in members of the genus *Brassica*, like cabbage, broccoli, cauliflower, Brussels sprouts, kale, and bok choy [1023]. 3,3′-diindolylmethane is a condensation product of indole-3-carbinol. Indole-3-carbinol and 3,3′-diindolylmethane are classified as blocking agents, and they are proposed as potential preventive agents against chronic disease including diabetes. Treatments with indole-3-carbinol and 3,3′-diindolylmethane increase the antioxidant-scavenging action by increasing levels of SOD, CAT, glutathione peroxidase (GPx), vitamin C, vitamin E, and glutathione in diabetic mice [1024].

Chlorogenic acid is a natural polyphenol found in many varieties of plant species. It stimulates glucose transport in skeletal muscle via AMPK activation. Chlorogenic acid has shown effects on hepatic glucose release and glycemia [1025,1026,1027].

Another natural phenol is ellagic acid; it is a dilactone acid found in fruits and vegetables. The antidiabetic effect of ellagic acid is attributed to the action on β cells of the pancreas that stimulates insulin secretion and decreases glucose intolerance. It possesses superior antioxidant properties, genotoxicity prevention, and α-amylase-inhibitory activity. Ellagic acid reduced hyperglycemia and insulin resistance in T2DM [1028,1029,1030].

Embelin is a hydroxyl benzoquinone found in *Embelia ribes*, *Lysimachia punctata*, and *Lysimachia erythrorhiza* species. It reduces the elevated plasma glucose, glycosylated hemoglobin, and pro-inflammatory mediators (interleukin 6 and tumor necrosis factor α) [1031,1032].

Erianin is a natural phenolic compound with 4 aromatic ether groups isolated from *Dendrobium chrysotoxum*. It inhibits high glucose-induced retinal angiogenesis via blocking the ERK1/2-regulated HIF-1α-VEGF/VEGFR2 signaling pathway [1033].

Gambogic acid (syn. guttic acid, guttatic acid, β-guttilactone, and β-guttiferin) is a natural pyranoxanthone; it is found in *Garcinia* plant species (*Garcinia hanburyi*, *Garcinia indica*, and *Garcinia cambogia*). It ameliorates diabetes-induced proliferative retinopathy through inhibition of the HIF-1α/VEGF expression via targeting the PI3K/AKT pathway [1034].

Garcinol is polyisoprenylated benzophenone found in a *Garcinia* species plant (*Garcinia indica*). It decreases plasma insulin, homeostasis model assessment of β-cell function (HOMA-β-cell) functioning index, glycogen, high-density lipoprotein cholesterol, body weight, and antioxidant enzyme activities. Garcinol reduces elevated levels of blood glucose, glycosylated hemoglobin, and lipids [1035,1036].

Honokiol is a polyphenol lignan predominantly found in *Magnolia* plant species (*Magnolia officinalis*). It increases phosphorylations and downstream insulin signaling factors. Honokiol showed potential binding mode to PTP1B [1037,1038]. Recently, Li et al. [1039] showed how honokiol protects pancreatic β cell against high glucose and intermittent hypoxia-induced injury by activating the Nrf2/ARE pathway in vitro and in vivo Withanolidesare isolated from *Withania somnifera.* They are found in plant sources from the Dioscoreaceae, Fabaceae, Lamiaceae, Myrtaceae, and Taccaceae families. Withanolides exhibited hypoglycaemic and hypolipidaemic activities [1040].

In conclusion, sources, structure, and target of 38 phytochemicals are summarised as potential antidiabetic agents. Most of the reviewed phytochemicals belong to flavonoids, alkaloids, and triterpenoids.

## 7. In Human Evidence: Clinical Studies

Currently, available conventional therapies for diabetes are challenged by their inherent limitations and medicinal plants are being researched as a source of alternative therapies [1053]. Of note, medicinal plants have been described in traditional medicine for the treatment of diabetes and have been experimentally shown to have, with their active constituents, antihyperglycemic or antidiabetic activity [1054]. However, information about their trials in humans is poorly documented. We describe in this section human clinical trials of medicinal plants for their antihyperglycemic or antidiabetes efficacy, including *Aloe vera*, *Cinnamomum burmanni*, *Cinnamomum cassia*, *Cinnamomum verum*, *Ginkgo biloba*, *Juglansregia*, *Malvastrumcoromandelia*, *Tinosporacordifolia*, *Trigonella foenum-graecum*, *Vitis vinifera*, and *Zingiber officinale.*

### 7.1. Aloe vera *(Asphodelaceae)*

Different types of *Aloe vera* extracts has been investigated in clinical trials. Four studies have been documented that involve prediabetic and T2DM patients (total N = 348) and that span between 6 to 8 weeks. The diabetic studies illustrated that *Aloe vera* significantly reduced FBG alone or in combination with the *Cnidoscoluschayamansa* extract. The *Aloe vera* juice (80%) investigated alongside glibenclamide in 72 T2DM patients (49 men and 23 women, aged 35–70 years, with high FBG levels and a typical diabetic curve of glucose tolerance analysis) did not show a response to glibenclamide alone while *Aloe vera* juice significantly reduced levels of FBG within two weeks and was safe on both kidney and liver [1055]. *Aloe vera* high-molecular-weight fractions (AHM) obtained from water-washed gel parts of *Aloe vera* leaves, cultivated in Okinawa, Japan and containing less than 10 ppm of barbaloin and polysaccharide (MW: 1000 kDa) with the glycoprotein virectin (MW: 29 kDa), produced a significant decrease in blood glucose levels sustained for six weeks from the start of the study. This study was performed on 15 T2DM patients (nine men and six women, aged 42–55 years, with FBG > 200 mg/dL). The treatment was safe on kidney and liver functions and was suggested to relieve vascular complications probably via activation of immunosystem [1056]. An *Aloe vera* (AG) gel complex (Aloe QDM complex) assessed in a randomized control trial showed borderline significant reductions in body weight, body fat mass, FBG, fasting serum insulin, and Homeostasis Model of Assessment-Insulin Resistance (HOMA-IR) after eight weeks of treatment [1057]. This study was performed on 136 patients with prediabetes or early T2DM not on medication (96 men and 40 women, aged ≥ 20 years, with body mass index (BMI) ≥ 25 kg/m^2^ or waist circumference ≥90 cm for men or ≥85 for women, FBG between 100 and 180 mg/dL or 2-h GTT ≥ 140 mg/dL, and HbA1c < 8.0%). To validate the antidiabetic claims for AG and infusion of *Cnidoscoluschayamansa* (CC) McVaugh, three double-blind crossover procedures were used in 125 women with early metabolic syndrome (mean age of 46.8 ± 9.7 years and waist circumference ≥ 88 cm, FGB ≥ 100 mg/dL, arterial blood preassure ≥ 130/≥ 85 mmHg, TG ≥ 150 mg/dL, and HDL < 50 mg/dL) [1058]. Assay 1: AG and CC vs. placebo 1 and placebo 2; assay 2: AG and placebo 2 vs. placebo 1 and CC; or assay 3: TA (total process *Aloe vera*, 5:1) vs. placebo 3. All combinations were tolerated except AG and P2 for which patient complained of bad taste and mild stomach pain because of the double dose of this treatment. Changes in HbA1c (mmol/mol) were assay 1: −1.8 ± 7.5 vs. −1.6 ± 6.9, *p* > 0.05; assay 2: −1.3 ± 6.6 vs. −1.4 ± 7.6, *p* > 0.05; and assay 3: −4.9 ± 8.3 vs. 0.44 ± 5.4, *p* < 0.01, respectively. TA concomitantly reduced high-sensitivity C-reactive protein (hs-CRP) (*p* < 0.05) and suggested that the total process *Aloe vera* decreases blood glucose levels by reducing proinflammatory state. The infusion of microwave-dehydrated *Cnidoscoluschayamansa* CC leaves did not reduce blood glucose or HDL and TG levels [1058].

### 7.2. Cinnamon: Cinnamomum cassia, Cinnamomum verum, Cinnamomum burmanni, Cinnamomum zeylanicum *(Lauraceae)*

Cinnamon has a long history as an antidiabetic spice. Research has shown that adding cinnamon to the diet can help to lower the glucose level, but results from trials involving cinnamon supplements are conflicting amongst patients with diabetes and insulin-resistant patients, particularly the ability to reduce blood glucose levels and to inhibit protein glycation [1059,1060]. A review of six trials investigating the potential benefit of cinnamon in controlling diabetes reveals contradicting findings in 178 diabetic or prediabetic patients. Oral administration in 79 patients with diagnosed T2DM (44 men and 21 women, under oral antidiabetics or diet) of the aqueous cinnamon purified extract 3 g/day for 4 months in a double-blind study significantly decreased the plasma glucose level (10.3%) compared to the placebo group (3.4%), supporting a moderate hypoglycemic effect of cinnamon [1061]. The combination of a water-soluble cinnamon bark extract (*Cinnamomumcassia* and/or *Cinnamomumburmanni* standardized to 3% Type A Polymers) administered (500 mg/day) for 12 weeks on twenty-two subjects with prediabetes and the metabolic syndrome was studied. Participants recruited for this study were between 30–60 years old, had FBG between 100 and 125 mg/dL, had BMI < 40 kg/m^2^, had normal values for liver and kidney function tests, and maintained their usual dietary and physical activity habits. The treatment significantly decreased FBG (−8.4%: 116.3 ± 12.8 mg/dL (pre) to 106.5 ± 20.1 mg/dL (post), *p* < 0.01) compared with the placebo group and suggests that cinnamon can reduce risk factors associated with diabetes and cardiovascular diseases [1062]. Gutierrez et al. [1063] found that a 5-g dose of *Cassia* cinnamon significantly reduces the blood glucose level and improves glucose tolerance following GTT by 10.1% with regards to the placebo groups in 10 sedentary and obese females (22.7 ± 4 years, BMI 35.39 ± 5.36 kg/m^2^). However, the treatment failed to improve insulin resistance and sensitivity [1063].

Though these results agree with the inability of cinnamon to improve insulin resistance or sensitivity, they are in contraction to its blood glucose lowering potency. Other studies showed that cinnamon supplementation (*Cinnamomum cassia*, 1.5 g/day) failed to improve whole-body insulin sensitivity or GTT in 25 postmenopausal patients with T2DM (aged 62.9 ± 1.5 years, BMI 30.4 ± 0.9 kg/m^2^) after six weeks [1064]. This finding is in line with that of Hasanzade et al. [1060], where cinnamon did not significantly affect FBG and glycosylated hemoglobin levels (*p* = 0.738 and *p* = 0.87, respectively) in a randomized clinical trial involving 70 T2DM (140 < FBG < 250 mg/dL; HbAlc > 7%) [1060]. Also, the administration of cinnamon (1 g/day) for 90 days in 72 adolescents with T1DM (diagnosis for ≥18 months before enrollment, aged 13–18 years) using a prospective, double-blind, placebo-controlled design did not improve glycemic control [1065]. This stresses the need to assess the real health benefits of cinnamon supplementation [1064]. However, most of these studies were conducted no longer than three months compared with the four months required for the mild antidiabetic potency reported.

### 7.3. Ginkgo biloba *(Ginkgoaceae)*

*Ginkgo biloba* is a popular medicinal plant used against metabolic syndromes and has been studied in humans for its ability to lower blood glucose. Three-month ingestion of a daily dose of 120 mg of *G. biloba* extract in normal glucose tolerant individuals (6 men and 14 women, aged 21–57 years) caused a significant increase in pancreatic β-cell insulin, fasting plasma insulin, and C-peptide response when compared to the placebo group [1066]. Following a 2-h standard GTT, glucose levels changed from 136 ± 55 to 162 ± 94 µU/mL/h *(p* = 0.1232) and 9.67 ± 5.34 to 16.88 ± 5.20 ng/mL/h (*p* < 0.001), respectively. However dissimilar insulin/C-peptide response curves were linked with an increased rate of insulin clearance induce by *G. biloba* [1066]. This finding was supported by the ability of *G. biloba* extract to affect the hypothalamic-pituitary-adrenal axis, leading to reduced basal cortisol levels and reduced cortisol production in response to the acute hyperglycemic challenge in 30 healthy/non-diabetic glucose tolerant volunteers (10 men and 20 women, 45.7 ± 9.9 years) in a randomized, double-blind, placebo-controlled crossover study. Fasting plasma cortisol was significantly lower after the *G. biloba* cycle than the placebo cycle (326 ± 149 vs. 268 ± 121 nmol/L, respectively; *p* = 0.19) [1067]. A follow-up study carried out in T2DM patients showed that, in diet-controlled subjects (FBG 117 ± 16 mg/dL; fasting plasma insulin 29 ± 8 µU/mL; *n* = 6), ingestion of *G. biloba* produced no significant effect on the insulin before and after ingesting *G. biloba*, respectively [1068]. However, in hyperinsulinemic T2DM patients, co-administration of oral hypoglycemic medications (*n* = 6) (FBG 143 ± 48 mg/dL; fasting plasma insulin 46 ± 13 µU/mL) and *G. biloba* caused blunted plasma insulin levels from 30 to 120 min during the GTT, leading to a reduction of the insulin area under the curve (AUC; 199 ± 33 vs 147 ± 58 µU/mL/h, before and after *G. biloba*, respectively) whereas the C-peptide levels did not increase in a parallel manner with the insulin, indicating an enhanced hepatic extraction of insulin relative to C-peptide as previously reported in normal glucose tolerant individuals. This suggests that ingestion of *G. biloba* in individuals with maximally stimulated pancreatic β cells may lead to a reduction in plasma levels of insulin. However, T2DM patients with pancreatic exhaustion (FPG 152 ± 46 mg/dL; fasting plasma insulin 16 ± 8 µU/mL; *n* = 8), treated as above, showed a significant increase in pancreatic β-cell function in response to glucose loading (insulin AUC increased from 51 ± 29 to 98 ± 20 µU/mL/h, *p* < 0.0001), paralleled by a C-peptide AUC increase from 7.2 ± 2.8 to 13.7 ± 6.8 (*p* < 0.0001). The authors linked this effect to a plausible increase of the activity in the remaining functional islets or to a regeneration of previously exhausted islets. According to this study, the ingestion of *G. biloba* extract by T2DM patients may increase the hepatic metabolic clearance rate of not only insulin but also the hypoglycemic agents and, thereby, may reduce insulin-mediated glucose metabolism and elevated blood glucose [1068].

### 7.4. Juglans regia *(Juglandaceae)*

The *Juglans regia* leaf has been traditionally used for the treatmen of DM in Iran, and its effects on hyperglycemia and lipid profiles have been investigated in 61 T2DM patients [1069]. Select patients with FBG between 150 and 200 mg/dL, glycated hemoglobin (HbA1c) between 7% and 9% and aged between 40 and 60 years were randomly divided into *J. regia* and placebo treatment groups. *J. regia* treatment, with 100-mg capsules administered thrice a day for three months along with the standard anti-diabetic therapy (metformin and glibenclamide, and nutritional regimen), improves glucose control by significantly decreasing the FBG, HbA1c, TC, and TG levels compared to placebo and did not affect liver and kidney but rather showed gastrointestinal disorder [1069].

### 7.5. Malvastrum coromandelianum *(Malvaceae)*

The water extract from *Malvastrum coromandelianum* has been shown to have a glucose-lowering effect and short- and long-term safety in animal studies. A study in humans reveals its safety and the poor glycemic-lowering efficacy of *M. coromandelianum* in T2DM subjects. Tharavanij et al. [1070] conducted a multicenter randomized, double-blind, placebo-controlled trial with 71 diabetes subjects under either diet control or single oral antidiabetic drug (sulphonylurea or biguanide) with HbA1C between 6.5–9.0%. Subjects received a tablet of 1200 mg/day of *M. coromandelianum* or placebo for 12 weeks. *M. coromandelianum* failed to significantly lower the blood glucose level and affect body weight, insulin resistance, and insulin secretion [1070].

### 7.6. Sauropus androgynus *(Phyllanthaceae)*

*Sauropus androgynus* is one of the most popular herbs in South Asia, Southeast Asia, and China, where it was known as a slimming agent and was identified to have antidiabetic activity [1071,1072]. A clinical trial corroborates this result and its use as an antidiabetic agent in the Ayurvedic medical system (*n* = 18 non-insulin-dependent diabetic, aged 50–65 years and weighted 70–85 kg) [1053]. *S*. *androgynus* (10 g/200 mL water) significantly reduces blood glucose level with glycemic index (GI) scores (GI = 55) lower than that of the glucose control (GI = 100). The hypoglycaemic activity of *S*. *androgynus* supports further investigation to unveil compounds/extracts with antidiabetic activity [1053].

### 7.7. Tinospora cordifolia *(Menispermaceae)*

Antidiabetic properties of *Tinospora cordifolia* are highly appreciated in *Ayurveda*, and studies on its extracts revealed its antihyperglycemic, preventive, and curative antidiabetic efficacy [633] in addition to its safety profile [1073]. From the three clinical studies reviewed here, 148 T2DM patients were involved in randomized control trials. *T. cordifolia* extracts were shown to lower FBG. The blood glucose-lowering effect of the aqueous leaf digest prepared from *T*. *cordifolia* (10 g/200mL water) was demonstrated using GTT. *T. cordifolia* was found to exhibit a significant ability to reduce blood sugar (GI = 39) levels compared to that of the glucose control (GI = 100) in human subjects (aged 50–65 years and weighted 70–85 kg), with the glucose levels reverting to fasting levels after 2 h of administration in the experimental groups [1053]. Additionally, the aqueous leaf digest (10 g/200 mL water) on post-prandial blood glucose levels in T2DM was found to exhibit a significant ability to reduce blood sugar levels in human subjects. Its hypoglycaemic potential was substantiated by a similar response observed in another study, wherein two extracts exerted significant hypoglycemic and antihyperglycaemic activity. However, solidified aqueous extract was shown to be more effective than sedimented starchy aqueous extract to control glycemic levels [633]. The hypoglycaemic effect of *T. cordifolia* and its healing efficacy in diabetic foot ulcers along with decoction for regular dressing was investigated on 60 patients suffering from uncontrolled T2DM patients with a diabetic foot ulcers. They received Ayurvedic oral hypoglycaemic drugs or insulin if needed. The aqueous extract of *T. cordifolia* stems soaked overnight and administered twice a day (30 mL) lowered blood sugar level along with other oral hypoglycaemic drugs. Deep root infection with variable blood sugar involving the bone tissue needs more than three months to heal with 80% of good healing without amputation. However, patients with established vascular changes with gangrenous toes (20%) needed a minor amputation of toes, but the ulcer was healed up very quickly with the same therapy [1074]. *T. cordifolia*, at a dose of 500 mg/day, is safe and improved living functions by regulating carbohydrate and lipid metabolism in 30 healthy individuals for 21 days [633,1075]. Moreover, Mishra et al. [1073] showed that *T. cordifolia*, at a dose of 500 mg three times daily, along with their conventional medications, was effective in decreasing the fasting and post-prandial blood glucose levels in patients with T2DM with no significant effect on the kidneys and liver [1073].

### 7.8. Trigonella foenum-graecum *(Fabaceae)*

*Trigonella foenum-graecum*, commonly known as fenugreek, is a plant that has been extensively used in cooking and as a source of antidiabetic compounds from its seeds and leaf extracts. There is evidence of its effectiveness in lowering postprandial glucose levels, but the long-term effect remains unclear [1076,1077]. Preliminary human trials and animal experiments suggest that orally administered *T. foenum-graecum* seed powder could have hypoglycaemic and antihyperlipidemic properties comparable to that of insulin [1076]. Results from clinical trials using FBG, 2 h GTT, and HbA1c and randomized models demonstrated the ability of fenugreek to significantly reduce both FBG and HbA1c in T2DM patients as compared with control interventions [1077,1078,1079,1080,1081,1082]. The effects of *T. foenum-graecum* seeds on glycemic control and insulin resistance, determined by the HOMA model, in mild to moderate T2DM showed that 1 g/day hydroalcoholic extract of fenugreek seeds improves glycemic control with antihypertriglyceridemic and decreases insulin resistance (25 newly diagnosed T2DM patients, FBG < 200 mg/dL) [1083]. However, different treatment regimens were used in each case, the clinical trial was poorly designed, and the results achieved cannot be conclusive and warrants further studies.

### 7.9. Vitis vinifera *(Vitaceae)*

*Vitis vinifera* grape polyphenols (2 g/day) investigated in 38 healthy overweight/obese first-degree relatives of T2DM patients (aged 30–65 years, BMI between 25 and 35 kg/m^2^, waist circumference >94 cm for men and >80 cm for women, FBG < 110 mg/dL) in a randomized, double-blind controlled trial demonstrated that grape polyphenols at nutritional doses effectively prevent fructose-induced oxidative stress and insulin resistance [1084].

### 7.10. Zingiber officinale *(Zingiberaceae)*

*Zingiber officinale* is a medicinal plant and spice extensively used in the control of diabetes. Arablouet et al. [1085] demonstrated that *Zingiber officinale* consumption in 70 T2DM patients (aged 30–70 years, BMI between 20 and 35 kg/m^2^, and HbA1C between 7 and 10%) significantly reduced FBG, HbA1C, insulin, HOMA, TG, TC, CRP, and PGE2 compared to the placebo group, suggesting an improvement of insulin sensitivity and the prevention of complications in T2DM patients [1085]. This result correlates with that obtained by Mahluji et al. [1086], where the administration of ginger 2 g/day for two months in a randomized double-blind placebo-controlled trial including 64 patients with T2DM (aged 38–65 years, average BMI of 29.5 kg/m^2^) significantly lowered the levels of insulin (11.0 ± 2.3 versus 12.1 ± 3.3; *p* = 0.001), LDL-C (67.8 ± 27.2 vs. 89.2 ± 24.9; *p* = 0.04), TG (127.7 ± 43.7 vs. 128.2 ± 37.7; *p* = 0.03) and the HOMA index (3.9 ± 1.09 vs. 4.5 ± 1.8; *p* = 0.002) and increased the quantitative insulin-sensitivity check index (0.313 ± 0.012 vs. 0.308 ± 0.012; *p* = 0.005) in comparison to the control group [1086]. These achieved results support the use of ginger to control hyperglycemia.

### 7.11. DBCare^®^ (Ace Continental Exports Inc., London, UK)

DBCare^®^ is a traditional herbal food supplement marketed as an antidiabetic medicine composed of 11 herbal ingredients. DBcare investigation in 35 patients with T2DM under oral hypoglycemic treatment (20 male and 15 women, HbA1C > 7.0%) showed safety and seems to decline the level of HbA1C (0.4 ± 0.7% in the DBCare^®^ group and 0.2% ± 0.8% in the placebo group; *p* = 0.806). However, no significant change was found in the fasting plasma glucose throughout the 12-weeks randomized, double-blind placebo-controlled trial, except episodic hypoglycemic effects observed in two patients. Though DBcare poorly controls blood glucose, a further study involving patients with HbA1C ≥ 8%, short (≤10 year) duration of diabetes, or young age, in particular, is commendable [1087].

## 8. Conclusions

The present review attempts to be useful to the scholars, scientists, and health professionals working in the field of pharmacology and therapeutics to develop antidiabetic drugs. In this work, we discussed traditional medicinal plants for the treatment of DM. Several plants with antidiabetic, antihyperglycemic, and hypoglycemic activities and with α-amylase and α-glucosidase inhibition are reported. The antidiabetic effect of plants is attributed to the mixture of phytochemicals or single components of the plant extracts. The phytochemicals responsible for antidiabetic properties mainly are alkaloids, phenolic acids, flavonoids, glycosides, saponins, polysaccharides, stilbenes, and tannins. In the several animal studies reported using different plants, there is a wide variety between the extraction methods, which is determinant in the phytochemical composition of the extracts. Moreover, phytochemical plant composition is highly dependent on several endogenous and exogenous factors, including genetic traits; plant organs used; and the growing, drying, and storing conditions. Stress factors, such as adverse climatology, and diseases affecting the plant also influence the phytochemicals obtained. Notwithstanding, these studies are still useful to discover a new natural antidiabetic drug which could be a great promise. As was discussed, low efficacy and safety concerns of current antidiabetic drugs of hundreds of millions of individuals have resulted in a current top-priority health-issue-seeking better management of diabetes.

Diverse mechanisms are described, explaining the beneficial effects of phytochemicals, such as regulation of glucose and lipid metabolism, insulin secretion, stimulating β cells, NF-*k*B signalling pathway, inhibition of gluconeogenic enzymes, and ROS protective action. In this relation, the investigation of phytochemicals responsible for the antidiabetic effects have progressed in the last few decades. Treating DM with plant-derived compounds, which are accessible and do not require laborious pharmaceutical synthesis, seems highly attractive.

Advances in traditional medicine research have significantly fuelled the drug development of novel entities for diabetes. It is worth noting that only a few medicinal plants have been studied for efficacy in humans. The majority of the reports failed to provide the authority name of herbs, the composition of the formulation, and preparation procedures. Most methods used for clinical trials were poorly designed, leading mostly to inconclusive findings. Therefore, more efficient clinical studies are warranted for further validation. On the other hand, efforts should be made to characterize antidiabetic active principles from antidiabetic plants. Moreover, as future perspectives, the medicinal plants described may be useful in the design of new functional foods with antidiabetic properties or for avoiding hyperglycemic effects of some foods like those rich in simple carbohydrates.

## Figures and Tables

**Table 1:** Antidiabetic plants.

Genus	Species	Geographic Zone	Activity	Reference
*Acacia*	*Acacia nilotica*		antidiabetic	[148]
	*Acacia catechu*	Nepal, India	antihyperglycemic	[149,150,151]
	*Acacia farnesiana*	Bangladesh	antidiabetic	[133,152]
	*Acacia tortilis*		antidiabetic	[153]
	*Acacia senegal*	Sudan	antidiabetic	[154]
	*Acacia ferruginea*		antidiabetic	[155]
	*Acacia nilotica*		antidiabetic	[156]
	*Acacia modesta*	India and Pakistan	antihyperglycemic	[157]
	*Acacia arabica*	India	hypoglycemic and antihyperglycemic	[158]
*Acalypha*	*Acalypha indica*	India	antidiabetic	[135,159]
	*Acalypha langiana*		antidiabetic	[160]
	*Acalypha wilkesiana*	Nigeria	antidiabetic	[161]
*Acanthopanax*	*Acanthopanax gracilistylus*	Korea	antidiabetic	[162]
	*Acanthopanax koreanum*	Korea	antidiabetic	[163]
	*Acanthopanax senticosus*	China (TCM)	antidiabetic	[164]
	*Acanthopanax sessiliflorus*	Southeast Asia	antidiabetic	[165]
*Achillea*	*Achillea millefolium*	India	antidiabetic	[151,166]
	*Achillea santolina*	Iraq and Jordan	antidiabetic	[167,168]
*Alisma*	*Alisma orientale*	China	antidiabetic	[169]
	*Alisma orientale*	China	hypoglycemic	[170]
*Allium*	*Allium ampeloprasum*	Iran	antidiabetic	[171]
	*Allium cepa*	Mauritius, Algeria	antihyperglycemic	[172,173,174,175]
	*Allium porrum*	Turkey	hypoglycemic	[176]
	*Allium sativum*	India (Ayurveda), Indonesia, Iran, Cuba, Mauritius, Togo, China (TCM)	α-amylase inhibitor, hypoglycemic, α-glucosidase inhibitor, antihyperglycemic	[128,173,175,177,178,179,180,181]
	*Allium stipitatum*	Iran	hypoglycemic, α-glucosidase inhibitor	[178]
*Aloe*	*Aloe ferox*	India (Ayurveda)	antidiabetic	[182]
	*Aloe marlothii*	South Africa	antidiabetic	[183]
	*Aloe vera*	India (Ayurveda), Ghana, Mauritius, Uganda, Tanzania, Traditional Chinese medicines, Trinidad and Tobago, Iran, Pakistan, Philippines, Saudi Arabia	α-amylase inhibitor, hypoglycemic	[52,61,63,128,138,181,184,185,186,187,188,189,190]
*Alpinia*	*Alpinia calcarata*	India, Sri Lanka	antidiabetic	[191,192]
	*Alpinia galanga*	India	antidiabetic	[193]
	*Alpinia officinarum*	China	hypoglycemic	[109]
*Amaranthus*	*Amaranthus cruentus*	Kenya	antidiabetic	[194]
	*Amaranthus hybridus*	Mauritius	antidiabetic	[186]
	*Amaranthus spinosus*	Taiwan	α-glucosidase inhibitor	[195,196]
*Angelica*	*Angelica hirsutiflora*	Taiwan	antidiabetic	[197]
	*Angelica keiskei*	Japan	antidiabetic	[198]
	*Angelica sinensis*	China (TCM)	antidiabetic	[199]
*Aralia*	*Aralia cachemirica*		antidiabetic	[200]
	*Aralia cortex*		antidiabetic	[201]
	*Aralia elata*	China, Korea, Japan	α-glucosidase inhibitor	[146,202]
	*Aralia taibaiensis*	China	α-glucosidase and α-amylase inhibitor	[203,204]
*Artemisia*	*Artemisia absinthium*		antidiabetic	[120,205]
	*Artemisia afra*	Africa	antidiabetic	[121]
	*Artemisia campestris*	Morocco	antidiabetic	[206]
	*Artemisia capillaris*		antidiabetic	[207]
	*Artemisia dracunculus*		antidiabetic	[208]
	*Artemisia judaica*	Jordan	antidiabetic	[209]
	*Artemisia herba-alba*	Iraq, Algeria, Jordan	hypoglycemic	[122,123,210]
	*Artemisia ludoviciana*	Mexico	hypoglycemic	[211]
	*Artemisia pallens*		antidiabetic	[212]
	*Artemisia parviflora*	India	antidiabetic	[213]
	*Artemisia princeps*	Asia	antidiabetic	[214]
	*Artemisia roxburghiana*		antidiabetic	[215]
	*Artemisia sacrorum*	China	antidiabetic	[216]
*Artocarpus*	*Artocarpus altilis*	Indonesia, Trinidad and Tobago, Mauritius	antidiabetic	[186,189,217]
	*Artocarpus communis*	Nigeria	antidiabetic	[218]
	*Artocarpus heterophyllus*	India (Ayurveda), Mauritius	hypoglycemic, α-amylase inhibitor	[186,219,220]
	*Artocarpus mariannensis*	Marshall Islands	antidiabetic	[221]
*Astragalus*	*Astragalus complanatus*	China	antidiabetic	[221]
	*Astragalus membranaceus*	China	antidiabetic	[222]
	*Astragalus propinquus*	China	α-glucosidase inhibitor	[223]
*Averrhoa*	*Averrhoa bilimbi*		antidiabetic	[224]
	*Averrhoa carambola*	Bangladesh	antihyperglycemic	[116]
*Berberis*	*Berberis aristata*	India (Ayurveda)	antidiabetic	[225,226]
	*Berberis asiatica*	India	antidiabetic	[227]
	*Berberis vulgaris*	Iran, China	antidiabetic	[228,229]
*Brassica*	*Brassica juncea*	India (Ayurveda)	antidiabetic	[172]
	*Brassica oleracea*		antihyperglycemic	[175]
	*Brassica rapa*	India	antidiabetic	[229]
*Buddleja*	*Buddleja asiatica*	India	antidiabetic	[230]
	*Buddleja cordata*	Mexico	antidiabetic	[231]
	*Buddleja officinalis*	Korea	antidiabetic	[232]
*Butea*	*Butea monosperma*	India	antidiabetic	[151]
	*Butea frondosa*	India	antidiabetic	[233]
*Caesalpinia*	*Caesalpinia bonducella*	India	α-amylase inhibitor	[234]
	*Caesalpinia ferrea*	Brazil	antidiabetic	[235]
*Calamus*	*Calamus tenuis*	India	antidiabetic	[125]
	*Calamus erectus*	India	antidiabetic	[236]
*Calotropis*	*Calotropis gigantea*	Bangladesh	antihyperglycemic	[237]
	*Calotropis procera*		antidiabetic	[238]
*Capparis*	*Capparis aphylla*		antihyperglycemic	[239]
	*Capparis decidua*	India, Pakistan	antidiabetic	[240,241]
	*Capparis sepiaria*	India	antidiabetic	[242]
	*Capparis spinosa*	India (Ayurveda and Unani)	antidiabetic	[243]
*Caralluma*	*Caralluma adscendens*	India	antidiabetic	[244,245]
	*Caralluma umbellata*	India	antihyperglycemic	[246]
*Carissa*	*Carissa carandas*	India (Ayurveda, Unani, and Homoeopathy)	antidiabetic	[247]
	*Carissa spinarum*	Kenya	antidiabetic	[248]
*Cassia*	*Cassia auriculata*	India, Tanzania	antidiabetic	[249,250]
	*Cassia fistula*	India	antidiabetic	[251]
	*Cassia obtusifolia*	China	antidiabetic	[252]
	*Cassia sieberiana*	Nigeria	antidiabetic	[253]
	*Cassia spectabilis*	Diabetes	antidiabetic	[254]
*Centaurea*	*Centaurea karduchorum*	Turkey	antidiabetic	[255]
	*Centaurea repens*	Persia	antidiabetic	[256]
	*Centaurea virgata*	Turkey	antidiabetic	[257]
*Cichorium*	*Cichorium pumilum*	Jordan	antidiabetic	[258]
	*Cichorium intybus*	Turkey	antidiabetic	[259]
*Cinnamomum*	*Cinnamomum burmannii*		antidiabetic	[260]
	*Cinnamomum cassia*	India (Unani, Ayurveda) Japan, China, South Africa	antidiabetic	[261,262]
	*Cinnamomum impressinervium*	India	antidiabetic	[104]
	*Cinnamomum iners*	Malaysia	antidiabetic	[263]
	*Cinnamomum japonicum*	Korea	antidiabetic	[264]
	*Cinnamomum obtusifolium*	Bangladesh	antidiabetic	[133]
	*Cinnamomum tamala*	India (Ayurveda)	hypoglycemic	[113]
	*Cinnamomum verum*	India (Ayurveda)	α-amylase inhibitor	[128]
	*Cinnamomum zeylanicum*		α-glucosidase	[147,265]
*Cistus*	*Cistus laurifolius*	Turkey	antidiabetic	[266]
	*Cistus ladaniferus*	Morocco	antidiabetic	[267]
	*Cistus monspeliensis*	Morocco	antidiabetic	[268]
	*Cistus salviifolius*	Morocco	antidiabetic	[268]
*Citrus*	*Citrus aurantium*		antidiabetic	[269]
	*Citrus grandis*	China	antidiabetic	[270]
	*Citrus paradisi*	Nigeria, Cuba, Trinidad and Tobago	antidiabetic	[179,189,271]
	*Citrus reticulata*	China	antidiabetic	[199]
	*Citrus sinensis*	India	antidiabetic	[272]
*Clerodendrum*	*Clerodendrum glandulosum*	India	antidiabetic	[273]
	*Clerodendrum colebrookianum*	India	antidiabetic	[230]
	*Clerodendrum capitatum*	Africa	antidiabetic	[274]
	*Clerodendrum inerme*		antidiabetic	[275]
	*Clerodendrum infortunatum*	India	antidiabetic	[276]
	*Clerodendrum phlomidis*	India (Ayurveda)	antidiabetic	[277]
*Coccinia*	*Coccinia cordifolia*	India	antidiabetic	[278]
	*Coccinia grandis*	India (Ayurveda), Sri Lanka	antihyperglycemic, α-glucosidase inhibitor, α-amylase inhibitor	[128,279,280,281]
	*Coccinia indica*	India (Ayurveda)	antidiabetic	[113,172]
*Coptis*	*Coptis chinensis*	China	antidiabetic	[282]
	*Coptis deltoidea*	China	antidiabetic	[282]
	*Coptis japonica*	China	antidiabetic	[282]
*Cordyceps*	*Cordyceps sinensis*	China	antidiabetic	[283]
	*Cordyceps militaris*		antidiabetic	[284]
*Cornus*	*Cornus officinalis*	China	antidiabetic, α-glucosidase inhibitor	[285,286]
	*Cornus kousa*	China	antidiabetic	[287]
	*Cornus mas*	China	antidiabetic	[288]
	*Cornus nuttallii*	Canada	antidiabetic	[289]
	*Cornus stolonifera*	Canada	antidiabetic	[290]
*Costus*	*Costus igneus*	India	antidiabetic	[291]
	*Costus pictus*	India	antidiabetic	[141]
	*Costus speciosus*	Sri Lanka	antidiabetic	[279]
*Croton*	*Croton cajucara*		antidiabetic	[292]
	*Croton celtidifolius*	Brazil	antidiabetic	[293]
	*Croton guatemalensis*	Guatemala	antidiabetic	[124]
	*Croton klozchianus*	India (Ayurveda)	antidiabetic	[294]
	*Croton zambesicus*		antidiabetic	[295]
*Cucumis*	*Cucumis callosus*	India	antidiabetic	[296]
	*Cucumis sativus*	Malaysia	antidiabetic	[297]
*Cucurbita*	*Cucurbita ficifolia*	Iran, Mexico	hypoglycemic	[175,298,299,300]
	*Cucurbita pepo*	South Africa	antidiabetic	[262]
*Curculigo*	*Curculigo latifolia*		antidiabetic	[301]
	*Curculigo orchioides*	India (Ayurveda)	antidiabetic	[302]
	*Curculigo recurvata*	Bangladesh	antidiabetic	[133]
*Curcuma*	*Curcuma angustifolia*	India	antidiabetic	[303]
	*Curcuma domestica*	India	antidiabetic	[151]
	*Curcuma longa*	China, Bangladesh, India (Ayurveda), Indonesia, Laos	antidiabetic	[177,181,226,304,305,306]
	*Curcuma xanthorrhiza*	Bangladesh, Indonesia, Laos	antidiabetic	[306,307,308]
*Cuscuta*	*Cuscuta reflexa*	India, Bangladesh	antidiabetic	[125,126]
	*Cuscuta chinensis*	China	antidiabetic	[309]
	*Cuscuta americana*	Trinidad and Tobago	antidiabetic	[189]
*Cynomorium*	*Cynomorium coccineum*	Saudi Arabia, China, Afghanistan, Mongolia, Iran	antidiabetic	[310]
	*Cynomorium songaricum*	Saudi Arabia, China, Afghanistan, Mongolia, Iran	antidiabetic	[310]
*Cyperus*	*Cyperus kyllinga*	India (Ayurveda)	antidiabetic	[311]
	*Cyperus laevigatus*	India (Ayurveda)	antidiabetic	[312]
	*Cyperus rotundus*	India (Ayurveda)	antidiabetic	[313]
*Delonix*	*Delonix regia*	Bangladesh	antidiabetic	[314]
	*Delonix elata*		antidiabetic	[315]
*Dendrobium*	*Dendrobium nobile*	Korea	antidiabetic	[316]
	*Dendrobium loddigesii*	China	α-glucosidase inhibitor	[317]
*Desmodium*	*Desmodium gangeticum*	India (Ayurveda), Sri Lanka	antidiabetic	[279,318]
	*Desmodium gyrans*	China (TCM)	antidiabetic	[319]
	*Desmodium styracifolium*	China (TCM)	antidiabetic	[319]
*Dioscorea*	*Dioscorea alata*		antidiabetic	[320]
	*Dioscorea bulbifera*		α-amylase, α-glucosidase inhibitor	[321]
	*Dioscorea japonica*	Korea	antidiabetic	[322]
	*Dioscorea nipponica*	Korea	antidiabetic	[323]
	*Dioscorea opposita*	China, India (Ayurveda), China (TCM)	antidiabetic	[181,226,324]
*Diospyros*	*Diospyros canaliculata*	Cameroon	antidiabetic	[325]
	*Diospyros crassiflora*	Cameroon	antidiabetic	[325]
	*Diospyros lotus*		antidiabetic	[326]
	*Diospyros melanoxylon*	India, Sri Lanka	antidiabetic	[327]
	*Diospyros peregrina*	India	antidiabetic	[328]
*Elephantopus*	*Elephantopus scaber*	India	antidiabetic	[329]
	*Elephantopus mollis*		antidiabetic	[330]
*Embelia*	*Embelia madagascariensis*		hypoglycemic	[331]
	*Embelia ribes*	India (Ayurveda)	antidiabetic	[332]
*Enicostema*	*Enicostema axillare*	India (Ayurveda)	antidiabetic	[333]
	*Enicostema littorae*		antidiabetic	[334]
*Erica*	*Erica arborea*	Turkey	antidiabetic	[335]
	*Erica bocquetii*	Turkey	antidiabetic	[335]
	*Erica sicula*	Turkey	antidiabetic	[335]
*Erythrina*	*Erythrina indica*	India	antidiabetic	[336]
	*Erythrina variegeta*	India	antidiabetic	[315]
*Eucalyptus*	*Eucalyptus globulus*	Iran	antihyperglycemic	[337,338]
	*Eucalyptus torreliana*	Nigeria	antihyperglycemic	[339,340]
*Eugenia*	*Eugenia cumini*		α-amylase inhibitor	[127]
	*Eugenia jambolana*	India (Ayurveda)	α-amylase inhibitor	[172,341]
	*Eugenia polyantha*	India, Indonesia	antidiabetic	[96,144]
	*Eugenia uniflora*	Paraguay	α-glucosidase inhibitor	[342]
*Euonymus*	*Euonymus laxiflorus*	Vietnam	antidiabetic	[343]
	*Euonymus alatus*	China (TCM)	antidiabetic	[344]
*Euphorbia*	*Euphorbia caducifolia*	India	antidiabetic	[132]
	*Euphorbia dioeca*		α-glucosidase inhibitor	[345]
	*Euphorbia drumondii*	India (Ayurveda)	hyperglycemic	[136,346]
	*Euphorbia hirta*	India, Bangladesh, Nepal	α-glucosidase	[93,133,150,347]
	*Euphorbia humifusa*	Mongolia	antidiabetic	[60]
	*Euphorbia kansui*		antidiabetic	[134]
	*Euphorbia ligularia*	India	antidiabetic	[104]
	*Euphorbia neriifolia*	India (Ayurveda)	antidiabetic	[131]
	*Euphorbia prostrata*		antihyperglycemic	[348]
	*Euphorbia thymifolia*	Bangladesh	antihyperglycemic	[116]
*Ferula*	*Ferula assa-foetida*	India (Ayurveda), Iran, Afghanistan	antidiabetic	[349,350]
	*Ferula feruloides*	Mongolia	antidiabetic	[60]
	*Ferula hermonis*	Lebanon, Syria	antidiabetic	[351]
	*Ferula persica*	Jordan	hypoglycemic	[352]
*Ficus*	*Ficus amplissima*	India (Ayurveda, Siddha, Unani)	antidiabetic	[353]
	*Ficus benghalensis*	India (Ayurveda, Siddha, Unani, homoeopathy), Southeast Asia	antidiabetic	[114,354,355,356]
	*Ficus carica*	India (Ayurveda, Siddha, Unani, homoeopathy)	antidiabetic	[357,358]
	*Ficus cunia*	India	α-glucosidase inhibitor	[359]
	*Ficus deltoidea*	Malaysia, Southeast Asia	α-glucosidase inhibitor	[360,361,362]
	*Ficus elastica*	Philippines	antidiabetic	[62]
	*Ficus exasperata*	Nigeria, Cameroon, Ivory Coast, Sierra Leone	antidiabetic	[253,363]
	*Ficus glomerata*	India (Ayurveda, Siddha, Unani, homoeopathy)	antidiabetic	[113,364]
	*Ficus glumosa*	Nigeria, Cameroon	hypoglycemic	[365,366,367]
	*Ficus hispida*	Bangladesh	antihyperglycemic	[116,368]
	*Ficus lutea*	Africa	antidiabetic	[119]
	*Ficus microcarpa*	in south Asia	antidiabetic	[369,370]
	*Ficus palmata*		antidiabetic	[371]
	*Ficus racemosa*	India (Ayurveda, Siddha, Unani, homoeopathy), Bangladesh, Southeast Asia	antihyperglycemic, hypoglycemic, α-glucosidase and α-amylase inhibitor	[83,356,372,373,374,375,376]
	*Ficus religiosa*	India (Ayurveda)	antidiabetic	[354,377]
	*Ficus sansibarica*	Africa	antidiabetic	[378]
	*Ficus thonningii*	Africa	antidiabetic	[363]
	*Ficus virens*	India (Ayurveda)	antidiabetic	[379]
*Gardenia*	*Gardenia gasminoides*	China	antidiabetic	[380]
	*Gardenia ternifolia*	Togo	antidiabetic	[180]
*Gentiana*	*Gentiana crassicaulis*		antidiabetic	[366]
	*Gentiana scabra*	Korea	antidiabetic	[381]
*Geranium*	*Geranium dielsianum*		antidiabetic	[382]
	*Geranium graveolens*	Jordan	antidiabetic	[383]
*Glycyrrhiza*	*Glycyrrhiza glabra*	China, India	antidiabetic	[181,384]
	*Glycyrrhiza uralensis*	India	antidiabetic	[385]
*Grewia*	*Grewia asiatica*	India (Ayurveda)	antidiabetic	[386]
	*Grewia hirsuta*	India	antidiabetic	[387]
	*Grewia nervosa*		antidiabetic	[388]
*Gynura*	*Gynura divaricata*	China	antidiabetic	[389]
	*Gynura formosana*	China	antidiabetic	[390]
	*Gynura procumbens*	Indonesia, Malaysia, Thailand, Southeast Asia, Korea	antidiabetic	[391,392,393,394]
	*Gynura segetum*		antidiabetic	[395]
*Hedysarum*	*Hedysarum limprichtii*	China	antidiabetic	[396]
	*Hedysarum polybotrys*	China	antidiabetic	[396]
	*Hedysarum smithianum*	China	antidiabetic	[396]
	*Hedysarum vicioider*	China	antidiabetic	[396]
*Helichrysum*	*Helichrysum caespititium*	South Africa	antidiabetic	[183]
	*Helichrysum graveolens*	Turkey	α-amylase inhibitor	[142]
	*Helichrysum italicum*	Europe	antidiabetic	[397]
*Helicteres*	*Helicteres hirsuta*	Southeast Asia	antidiabetic	[398]
	*Helicteres isora*	India (Ayurveda)	antidiabetic	[399]
*Holarrhena*	*Holarrhena antidysenterica*	India (Ayurveda)	antidiabetic	[400]
	*Holarrhena floribunda*	Nigeria	α-amylase inhibitor	[401]
*Hydnocarpus*	*Hydnocarpus alpina*		hypoglycemic	[402]
	*Hydnocarpus wightiana*	India (Ayurveda)	antidiabetic	[403]
*Juniperus*	*Juniperus oxycedrus*	Turkey	α-amylase inhibitor, hypoglycemic activity	[142,404]
	*Juniperus communis*	Turkey	α-glucosidase inhibitor	[142]
*Justicia*	*Justicia adhatoda*	Pakistan	antidiabetic	[405]
	*Justicia gendarussa*		antidiabetic	[406]
	*Justicia secunda*		antidiabetic	[407]
	*Justicia spicigera*		antidiabetic	[408]
*Leucas*	*Leucas aspera*	India, Bangladesh	antidiabetic	[193,409]
	*Leucas cephalotes*	India (Ayurveda), Nepal, Pakistan	antidiabetic	[410]
*Liriope*	*Liriope platyphylla*	China	antidiabetic	[411]
	*Liriope spicata*	China	antidiabetic	[412]
*Lonicera*	*Lonicera caerulea*	northern Russia, China, Japan	antidiabetic	[413]
	*Lonicera japonica*	China	antidiabetic	[414]
*Luffa*	*Luffa acutangula*		antidiabetic	[415]
	*Luffa cylindrica*		antidiabetic	[416]
	*Luffa echinata*	India	antidiabetic	[417]
*Lycium*	*Lycium barbarum*	China	antidiabetic	[181,418]
	*Lycium chinense*	China	antidiabetic, antihyperglycemic	[418,419,420]
	*Lycium ruthenicum*	China	antidiabetic	[421]
*Mangifera*	*Mangifera indica*	India (Ayurveda), Nigeria	α-amylase inhibitor, antihyperglycemic	[128,422]
	*Mangifera mekongensis*	Vietnam	α-glucosidase inhibitor	[423]
*Marrubium*	*Marrubium alysson*		α-glucosidase inhibitor	[424]
	*Marrubium deserti*	Tunisia	antidiabetic	[425]
	*Marrubium radiatum*	Lebanon	α-amylase inhibitor	[137]
	*Marrubium vulgare*	Mexico, Jordan, Algeria	antidiabetic	[231,426,427]
*Melia*	*Melia azadirachta*	Mexico	antidiabetic	[231]
	*Melia dubia*	India	antidiabetic	[428]
	*Melia orientalis*	India (Ayurveda)	antidiabetic	[429]
*Mentha*	*Mentha arvensis*	India	antidiabetic	[151]
	*Mentha longifolia*	India	antidiabetic	[151]
	*Mentha piperita*		antidiabetic	[430]
*Mimosa*	*Mimosa invisa*	Nigeria	hypoglycemic	[431]
	*Mimosa pigra*	Bangladesh	antihyperglycemic	[432]
	*Mimosa pudica*	Sri Lanka, Thailand	hypoglycemic	[279,433]
*Mimusops*	*Mimusops elengi*	India (Ayurveda)	antidiabetic	[434]
	*Mimusops zeyheri*	South Africa	antidiabetic	[183]
*Momordica*	*Momordica balsamina*	South Africa	antidiabetic	[183]
	*Momordica charantia*	Philippines, Vietnam, Mauritius, Trinidad and Tobago, India (Ayurveda), Nigeria, Bangladesh, Taiwan, central America	α-amylase inhibitor, hypoglycemic, antihyperglycemic	[61,85,113,129,186,189,435,436,437,438,439]
	*Momordica cymbalaria*		antidiabetic	[440]
	*Momordica foetida*	South Africa	antidiabetic	[441]
	*Momordica grosvenori*	China (TCM)	antidiabetic	[442]
*Moringa*	*Moringa oleifera*	South Africa, Kenya, Mexico, India (Ayurveda), Nigeria, Mauritius, Senegal	hypoglycemic	[113,183,194,231,443,444,445]
	*Moringa peregrina*		antidiabetic	[446]
	*Moringa stenopetala*	Ethiopia	α-glucosidase inhibitor	[139,444]
*Morus*	*Morus alba*	Iran, Philippines, Trinidad and Tobago, India (Ayurveda), China (TCM), Pakistan, Korea, Chile	antidiabetic, hypoglycemic, α-glucosidase and α-amylase inhibition	[53,62,189,447,448,449,450,451,452,453]
	*Morus nigra*	Iran, Jordon	antidiabetic	[53,57]
*Mucuna*	*Mucuna gigantea*	India	antidiabetic	[454]
	*Mucuna pruriens*	India (Ayurveda)	antidiabetic	[172]
*Murraya*	*Murraya koenigii*	India (Ayurveda)	α amylase inhibitor, hypoglycemic effects, antihyperglycemic	[455,456,457,458,459]
	*Murraya panicutata*	Nigeria	α-glucosidase inhibitor	[339]
*Musa*	*Musa acuminata*		antidiabetic	[460]
	*Musa paradisiaca*		antidiabetic	[460]
	*Musa Sapientum*	India	antihyperglycemic	[348,461]
*Nymphaea*	*Nymphaea nouchali*	Bangladesh, India (Ayurveda)	antidiabetic	[133,462]
	*Nymphaea stellata*	India (Ayurveda)	α-glucosidase inhibitor, hypoglycemic, antihyperglycemic	[463,464,465]
*Ocimum*	*Ocimum campechianum*	Trinidad and Tobago	antidiabetic	[189]
	*Ocimum canum*	Ghana	lowers blood glucose	[466,467]
	*Ocimum gratissimum*	Bangladesh, Nigeria	hypoglycemic	[133,436,468]
	*Ocimum sanctum*	India (Ayurveda), China, Bangladesh	hypoglycemic	[469,470,471,472]
	*Ocimum tenuiflorum*	India (Ayurveda)	α-amylase inhibitor, hypoglycemic, antihyperglycemic	[128,473]
*Oplopanax*	*Oplopanax elatus*	China, Russia, and Korea	antidiabetic	[474]
	*Oplopanax horridus*		antidiabetic	[475]
*Origanum*	*Origanum onites*	Turkey	antidiabetic	[476]
	*Origanum vulgare*		antidiabetic	[477]
*Orthosiphon*	*Orthosiphon aristatus*		antidiabetic	[478,479]
	*Orthosiphon stamineus*	Indonesia and Malaysia	antidiabetic	[480]
*Otostegia*	*Otostegia persica*	Iran	antidiabetic	[481]
	*Otostegia integrifolia*		antidiabetic	[482]
*Oxalis*	*Oxalis corniculata*	India	antidiabetic	[151]
	*Oxalis griffithii*	India	antidiabetic	[125]
*Paederia*	*Paederia foetida*	China, Vietnam, India Japan	antidiabetic	[483]
	*Paederia scandens*	China, Vietnam, India, Japan	antidiabetic	[483]
*Paeonia*	*Paeonia lactiflora*	Korea, China, Japan	hypoglycemic	[484]
	*Paeonia suffruticosa*	China, Korea, Japan	antidiabetic	[471,485]
*Pandanus*	*Pandanus amaryllifolius*		antihyperglycemic	[486]
	*Pandanus fascicularis*	India (Ayurveda)	antihyperglycemic	[487]
	*Pandanus tectorius*		antidiabetic	[488]
*Panax*	*Panax ginseng*	Korea	antidiabetic	[489]
	*Panax notoginseng*	China	antihyperglycemic	[490,491]
	*Panax quinquefolius*		antidiabetic	[492]
*Phaleria*	*Phaleria cumingii*		antidiabetic	[493]
	*Phaleria macrocarpa*	Indonesia, Malaysia, Papua	α-glucosidase inhibitor	[494,495,496,497]
	*Phaleria nishidae*		antidiabetic	[498]
*Phyllanthus*	*Phyllanthus amarus*	Vietnam, India (Ayurveda, Siddha, Unani and homeopathy), Nigeria, Malaysia	α-glucosidase inhibitor, hypoglycemic, α-amylase inhibitor	[83,499,500,501,502]
	*Phyllanthus emblica*	Thailand, Southeast Asia, India (Ayurveda)	antidiabetic	[75,356,503]
	*Phyllanthus engleri*	Tanzania	antidiabetic	[504]
	*Phyllanthus fraternus*		antidiabetic	[505]
	*Phyllanthus gardnerianus*	India	antidiabetic	[506]
	*Phyllanthus niruri*		hypoglycemic	[507,508]
	*Phyllanthus urinaria*	Vietnam	α-glucosidase and α-amylase inhibitor	[83]
	*phyllanthus virgatus*		α-amylase inhibitor	[509]
	*Phyllanthus watsonii*		antidiabetic	[510]
*Physalis*	*Physalis angulata*	India	antidiabetic	[511]
	*Physalis minima*	India	antidiabetic	[193]
	*Physalis peruviana*	India	antidiabetic	[248]
*Piper*	*Piper angustifolium*	Latin America	antidiabetic	[512]
	*Piper betle*	Asia	hypoglycemic	[513,514,515]
	*Piper crocatum*		antihyperglycemic	[516]
	*Piper cubeba*		α-amylase and α-glucosidase	[517]
	*Piper guineense*	Nigeria	α-amylase inhibitor	[401]
	*Piper longum*	Bangladesh, India (Ayurveda)	antihyperglycemic	[305,518,519]
	*Piper nigrum*		α-amylase inhibitor, hypoglycemic	[128,226,520]
	*Piper sarmentosum*	South East Asia	antidiabetic	[521,522]
*Pistacia*	*Pistacia atlantica*	Jordan	hypoglycemic	[168,352]
	*Pistacia integerrima*		antidiabetic	[523]
*Plantago*	*Plantago asiatica*		antidiabetic	[524]
	*Plantago lanceolata*	Turkey	α-amylase and α-glucosidase inhibitor	[525]
	*Plantago ovata*	India	antidiabetic	[341]
*Plumeria*	*Plumeria alba*	Togo	antidiabetic	[526]
	*Plumeria obtusa*	South Africa	antidiabetic	[183]
	*Plumeria rubra*	India	α-amylase and α-glucosidase inhibitor	[517,527]
*Polygonum*	*Polygonum cuspidatum*	Japan, Korea, China	α-glucosidase inhibitor	[528,529]
	*Polygonum hydropiper*	India	antidiabetic	[230]
	*Polygonum multiflorum*	China, Asia, Europe, Africa	hypoglycemic	[530,531,532]
	*Polygonum senegalensis*		antidiabetic	[533]
*Psidium*	*Psidium cattleianum*	east Asia	antidiabetic	[534]
	*Psidium guajava*	Mauritius, Togo, Sri Lanka, central America, Japan, China (TCM), Papua New Guinea	antihyperglycemic, hypoglycemic	[173,180,279,438,535,536,537]
*Pterocarpus*	*Pterocarpus santalinus*	India (Ayurveda)	antidiabetic	[538]
	*Pterocarpus marsupium*	India	antidiabetic	[539]
	*Pterocarpus soyauxii*		antidiabetic	[540]
*Prunus*	*Prunus persica*	India	antidiabetic	[541]
	*Prunus capuli*	Peru	antidiabetic	[542]
	*Prunus emarginata*	Canada	antidiabetic	[289]
	*Prunus mume*	China	antidiabetic	[543]
*Pueraria*	*Pueraria lobata*	Korea, China (TCM)	antidiabetic, α-glucosidase inhibitor	[544,545,546,547]
	*Pueraria thomsonii*		antidiabetic	[548]
	*Pueraria thunbergiana*	Korea	antidiabetic	[549]
*Rheum*	*Rheum emodi*	India (Ayurveda), China	antidiabetic	[550]
	*Rheum officinale*	China	antidiabetic	[551]
	*Rheum palmatum*	China	antidiabetic	[552]
	*Rheum ribes*	Iran, Jordon	hypoglycemic	[52,553,554]
	*Rheum tanguticum*	China	antidiabetic	[552]
	*Rheum turkestanicum*	Iran	antidiabetic	[555]
	*Rheum undulatum*	Korea	antidiabetic	[556]
*Rhododendron*	*Rhododendron brachycarpum*	Korea	antidiabetic	[557,558]
	*Rhododendron groenlandicum*		antidiabetic	[559]
	*Rhododendron tomentosum*	Canada	antidiabetic	[560]
*Rhus*	*Rhus coriaria*	Iran	antidiabetic	[561]
	*Rhus chinensis*		antidiabetic	[562]
	*Rhus hirta*		antidiabetic	[290]
	*Rhus mysorensis*		antidiabetic	[563]
	*Rhus verniciflua*	Korea	antidiabetic	[564]
	*Rhus virens*	Mexico	antidiabetic	[231]
*Rosa*	*Rosa canina*	Iran, Turkey	antidiabetic	[565,566]
	*Rosa rugosa*	Korea, China	hypoglycemic	[109,567,568]
*Salacia*	*Salacia chinensis*	India (Ayurveda, Unani), Japan, Korea	hypoglycemic, antihyperglycaemic	[569,570,571]
	*Salacia oblonga*	India (Ayurveda, Unani), Japan, Korea	hypoglycemic	[569,570,572]
	*Salacia prinoides*	India (Ayurveda), Sri Lanka, Southeast Asia	antidiabetic	[573]
	*Salacia reticulata*	India (Ayurveda, Unani), Japan, Korea, Sri Lanka	hypoglycemic, α-glucosidase inhibitor	[569,570,574,575]
*Salvia*	*Salvia acetabulosa*	Lebanon	α-amylase inhibitor	[137]
	*Salvia hispanica*	Central and South America	antidiabetic	[576]
	*Salvia hypoleuca*	Iran	antidiabetic	[577]
	*Salvia officinalis*	Iran	hypoglycemic, α-glucosidase inhibitor	[178]
	*Salvia libanotica*		antidiabetic	[578]
	*Salvia limbata*	Turkey	α-amylase and α-glucosidase inhibitor	[525]
	*Salvia miltiorrhiza*	China	antidiabetic	[181,579]
*Sida*	*Sida acuta*	India	antidiabetic	[580]
	*Sida cordifolia*	Bangladesh, India (Ayurveda)	antidiabetic	[471,581]
	*Sida rhombifolia*		antidiabetic	[582]
*Smilax*	*Smilax china*	Korea	antidiabetic	[583]
	*Smilax glabra*	China	antidiabetic	[584]
	*Smilax officinalis*	Latin America	antidiabetic	[512]
	*Smilax perfoliata*	Bangladesh	antihyperglycemic	[585]
*Solanum*	*Solanum americanum*	Guatemala	antidiabetic	[124]
	*Solanum indicum*	Uganda, India	antidiabetic	[104,187]
	*Solanum lycocarpum*	Brazil	antidiabetic	[586]
	*Solanum muricatum*		antidiabetic	[587]
	*Solanum nigrum*	Asia	hypoglycemic	[588,589]
	*Solanum torvum*		antihyperglycemic	[590]
	*Solanum trilobatum*	India (Ayurveda, Siddha)	antidiabetic	[118]
	*Solanum tuberosum*		antidiabetic	[591]
	*Solanum viarum*	India	antidiabetic	[125]
	*Solanum virginianum*	Pakistan	antidiabetic	[592]
	*Solanum xanthocarpum*		hypoglycemic	[593]
*Spondias*	*Spondias mombin*	Nigeria	α-amylase inhibition, hypoglycemic	[594]
	*Spondias pinnata*	Indonesia, Sri Lanka	antihyperglycemic	[595,596]
*Stereospermum*	*Stereospermum colais*		α-glucosidase inhibitor	[597]
	*Stereospermum suaveolens*	India	antidiabetic	[598]
*Swertia*	*Swertia chirata*	Bangladesh	antidiabetic	[126]
	*Swertia chirayita*	India (Ayurveda)	hypoglycemic	[113,599]
	*Swertia cordata*		antidiabetic	[600]
	*Swertia longifolia*		α-amylase inhibitor	[601]
	*Swertia macrosperma*	Tibet, China	antidiabetic	[602]
	*Swertia mussotii*	China	α-glycosidase inhibitor	[603]
*Syzygium*	*Syzygium alternifolium*		antidiabetic	[604]
	*Syzygium aromaticum*		antihyperglycemic, hypoglycemic	[605]
	*Syzygium cumini*	Bangladesh, India (Ayurveda), Brazil	α-glucosidase and α-amylase inhibitor, antihyperglycemic	[83,172,220,376,606,607,608]
	*Syzygium densiflorum*	India	antidiabetic	[609]
	*Syzygium jambolanum*	India (Ayurveda)	hypoglycemic	[610,611]
	*Syzygium jambosa*	Puerto Rico	hypoglycemic	[612]
	*Syzygium samarangense*	Bangladesh	antihyperglycemic	[116]
*Tabernaemontana*	*Tabernaemontana corymbosa*	Malaysia	antidiabetic	[613]
	*Tabernaemontana divaricata*	India	antidiabetic	[104]
	*Tabernaemontana heyneana*		antidiabetic	[614]
*Taxus*	*Taxus baccata*	India	antidiabetic	[151]
	*Taxus yunnanensis*	China	antidiabetic	[615]
*Terminalia*	*Terminalia alata*	Vietnam	antidiabetic	[616]
	*Terminalia arjuna*	Bangladesh, India (Ayurveda)	α-amylase inhibitor, antihyperglycemic	[126,127,617,618]
	*Terminalia bellirica*	Bangladesh, Vietnam, India (Ayurveda, Siddha, Unani), Sri Lanka, Southeast Asia	antidiabetic	[133,616,619,620]
	*Terminalia catappa*		antidiabetic	[621]
	*Terminalia chebula*	Thailand, India (Ayurveda), Bangladesh, Iran	α-amylase inhibitor	[75,128,130,622,623]
	*Terminalia citrina*	Bangladesh	antidiabetic	[133]
	*Terminalia corticosa*	Vietnam	antidiabetic	[616]
	*Terminalia glaucescens*	Cameroon	antidiabetic	[624]
	*Terminalia macroptera*	Africa	α-glucosidase inhibitor	[625]
	*Terminalia sericea*		antidiabetic	[626]
	*Terminalia superba*		antidiabetic	[627]
*Teucrium*	*Teucrium oliverianum*		antidiabetic	[628]
	*Teucrium polium*	Jordan, Iran	hypoglycemic	[553,629,630]
*Thymus*	*Thymus caramanicus*	Iran	antidiabetic	[631]
	*Thymus satureioides*	Morocco	antidiabetic	[632]
*Tinospora*	*Tinospora cordifolia*	Southeast Asia, India (Ayurveda), Thailand, Malaysia, Guyana, Bangladesh	α-amylase inhibitors, hypoglycemic, antihyperglycemic	[113,128,135,356,619,633,634,635]
	*Tinospora crispa*	Malaysia, Thailand, Malaysia, Guyana, Bangladesh, Indonesia, Malaysia	hypoglycemic, antihyperglycemic	[613,635,636,637,638,639,640]
	*Tinospora malabarica*		antidiabetic	[641]
	*Tinospora sinensis*	Nepal, India	antidiabetic	[150,642]
	*Tinospora bakis*	Sudan	antidiabetic	[643]
*Trichosanthes*	*Trichosanthes cucumerina*	India (Ayurveda)	hypoglycemic	[113]
	*Trichosanthes dioica*	India (Ayurveda)	antidiabetic	[644]
	*Trichosanthes kirilowii*	China (TCM)	hypoglycemic, α-amylase inhibitor	[645,646]
	*Trichosanthes tricuspidata*		hyperglycemic	[647]
*Urtica*	*Urtica angustifolia*		hypoglycemic	[648]
	*Urtica dioica*	Kenya, Iran, Turkey	α-amylase inhibitor	[248,649,650,651]
	*Urtica urens*		antidiabetic	[652]
*Vaccinium*	*Vaccinium angustifolium*		antidiabetic	[653]
	*Vaccinium arctostaphylos*	Iran	α-amylase inhibitor	[654]
	*Vaccinium bracteatum*	China	antidiabetic	[655]
	*Vaccinium myrtillus*		antidiabetic	[656]
	*Vaccinium ovalifolium*		antidiabetic	[657]
	*Vaccinium uliginosum*		antidiabetic	[657]
	*Vaccinium vitis*		antidiabetic	[658]
*Withania*	*Withania coagulans*	India (Ayurveda), Pakistan	antihyperglycemic	[659,660,661]
	*Withania somnifera*	India (Ayurveda)	hypoglycemic	[96,662]
*Zanthoxylum*	*Zanthoxylum alatum*		antidiabetic	[663]
	*Zanthoxylum armatum*	India (Ayurveda)	antidiabetic	[251]
	*Zanthoxylum capense*	South African	antidiabetic	[664]
	*Zanthoxylum chalybeum*	Tanzania	antidiabetic	[188]
	*Zanthoxylum humile*	India (Ayurveda)	antidiabetic	[665]
*Zingiber*	*Zingiber officinale*	India (Ayurveda), Latin America Africa	α-amylase inhibitor, hypoglycemic	[113,128,512,666]
	*Zingiber striolatum*	China (TCM)	hypoglycemic	[667]
*Ziziphus*	*Ziziphus amole*		antidiabetic	[668]
	*Ziziphus jujuba*	Turkey	α glucosidase inhibitor	[76,669]
	*Ziziphus lotus*	Algeria	antidiabetic	[670]
	*Ziziphus mauritiana*	Southeast Asia, Mali	antidiabetic	[356,671]
	*Ziziphus mucronata*	Nigeria	antidiabetic	[672]
	*Ziziphus nummularia*	India	antidiabetic	[132]
	*Ziziphus oxyphylla*	Pakistan	antidiabetic	[673]
	*Ziziphus spina-christi*	Egypt	hypoglycemic and anti-hyperglycemic	[674]
	*Ziziphus xylopyrus*	India (Ayurveda), Pakistan, China	antidiabetic	[675]

TCM Traditional Chinese Medicine.

**Table 2:** Antidiabetic plants where only one species is available.

Plant Name	Country/Region	Activity	Reference
*Abrus precatorius*	India (Ayurveda, Unani, Siddha)	antidiabetic	[676]
*Acorus calamus*	India, Indonesia, America	α-glucosidase inhibitor	[93,677,678]
*Actinidia arguta*	Korea	antidiabetic	[679]
*Adansonia digitata*	India (Ayurveda)	α-amylase inhibitor	[128]
*Adiantum capillus-veneris*	India	antidiabetic	[151]
*Ageratum conyzoides*	Bangladesh	antidiabetic	[126]
*Agrimonia pilosa*	China	α-glucosidase inhibitor	[680]
*Ailanthus excelsa*	India	antidiabetic	[681]
*Alangium salvifolium*	India (Ayurveda)	hypoglycemic	[682,683]
*Alstonia scholaris*	India, Thailand	α-glucosidase inhibitor	[87,684]
*Amomum villosum*	China	antidiabetic	[109]
*Amygdalus lycioides*	Iran	antidiabetic	[685]
*Andrographis paniculata*	India (Ayurveda), Bangladesh, Nepal, Malaysia, Southeast Asia	antihyperglycemic	[126,150,356,686,687]
*Anemarrhena asphodeloides*	China	antidiabetic, α-glucosidase inhibitor	[181,688,689]
*Anethum graveolens*	Iran, Asia	antidiabetic	[690,691]
*Anogeissus acuminate*	Thailand	hypoglycemic	[433]
*Anthocephalus cadamba*	India (Ayurveda), Australia, China, Indonesia, Malaysia, Papua New Guinea, Philippines, Singapore, Vietnam	antidiabetic	[692]
*Aphanamixis polystachya*	India (Ayurveda)	antidiabetic	[693]
*Arctium lappa*	China	hypoglycemic	[694]
*Argyreia nervosa*	India (Ayurveda)	antidiabetic	[695]
*Asanadi gana*	India (Ayurveda)	antidiabetic	[696]
*Azadirachta indica*	India (Ayurveda), Nigeria, Pakistan, Mexico, Bangladesh, Nepal, Saudi Arabia, South East Asia, Mauritius, Malaysia, Indonesia	α-glucosidase and α-amylase inhibitor, hypoglycemic	[65,113,126,135,150,190,220,231,253,356,697,698,699]
*Barringtonia acutangula*	India (Ayurveda)	antidiabetic	[700]
*Basella rubra*	India	α-amylase inhibitor	[701]
*Begonia roxburghii*	India	antidiabetic	[125]
*Bergenia ciliata*	Nepal	α-glucosidase, α-amylase inhibitor	[702]
*Biophytum sensitivum*	Nepal	antidiabetic	[703]
*Blepharis molluginifolia*	India	antidiabetic	[704]
*Boerhavia diffusa*	India (Ayurveda)	antidiabetic	[226]
*Boswellia ovalifoliolata*	India	antidiabetic	[705]
*Caccinium myrtillus*	Europe	α-glucosidase inhibitor	[706]
*Cajanus cajan*	India (Ayurveda)	antidiabetic	[172]
*Callicarpa arborea*	India	antidiabetic	[125]
*Camellia sinensis*	Iran	α-amylase inhibitor	[651]
*Canna indica*		antidiabetic	[707]
*Cardia obaliqua*	Pakistan	antidiabetic	[708]
*Carthamus tinctorius*	Iran	α-glucosidase inhibitor	[709,710]
*Casia fistula*	India (Ayurveda)	α-amylase inhibitor	[128]
*Catharanthus roseus*	India (Ayurveda), South Africa, China, Malaysia, South East Asian Countries, South Africa, Trinidad, Tobago	α amylase inhibitor, antihyperglycemic, hypoglycemic	[113,189,234,356,711,712,713,714,715]
*Catunaregam tormentosa*	Thailand	hypoglycemic	[433]
*Cayratia trifolia*	India	antidiabetic	[716]
*Ceiba pentandra*	India, Nigeria	α-amylase inhibition, hypoglycemic, antihyperglycemic	[717,718,719]
*Celosia argentea*	China	antidiabetic	[720]
*Centella asiatica*	India (Ayurveda), Bangladesh, Malaysia, Laos, Southeast Asia	antidiabetic	[133,306,356,721,722]
*Centranthus longiflorus*	Turkey	antidiabetic	[723]
*Centratherum anthelminticum*	India (Ayurveda)	hypoglycemic	[580,724]
*Cerinthe minor*	Turkey	antidiabetic	[723]
*Chlorophytum borivilianum*	India (Ayurveda)	antidiabetic	[725]
*Cirsium japonicum*	Taiwan	antidiabetic	[726]
*Cistanche tubulosa*	China	antihyperglycemic	[727]
*Citrullus colocynthis*	Iran, Algeria, Southeast Asia	hypoglycemic	[356,728,729]
*Clinacanthus nutans*	Indonesia, Malaysia, Thailand	antidiabetic	[730,731]
*Clitoria ternatea*	India (Ayurveda)	α-glucosidase, α-amylase inhibitor hypoglycemic	[452,732,733]
*Cocculus hirsutus*	India	α-amylase inhibitor	[701]
*Coldenia procumbens*	India	antidiabetic	[734]
*Commiphora wightii*	India (Ayurveda)	antidiabetic	[226]
*Coscinium fenestratum*	India, Sri Lanka	antidiabetic	[735,736]
*Cressa cretica*	Bahrain	antidiabetic	[737]
*Crossostephium chinense*	China	antidiabetic	[289]
*Cuminum cyminum*	India	antidiabetic	[738]
*Cupressus sempervirens*	Cyprus	antidiabetic	[739]
*Cyamopsis tetragonoloba*	India (Ayurveda)	antidiabetic	[740]
*Cyclocarya paliurus*	China	antidiabetic	[741]
*Cydonia oblonga*	Turkey	hypoglycemic	[176]
*Dendrocalamus hamiltonii*	India (Ayurveda)	hypoglycemic	[113]
*Dendrophthoe pentandra*	Indonesia	antidiabetic	[742]
*Desmostachya bipinnata*	India (Ayurveda)	antidiabetic	[743]
*Dillenia indica*	India	antidiabetic	[125]
*Dioecrescis erythroclada*	Thailand	hypoglycemic	[433]
*Diplazium esculentum*	India	antidiabetic	[125]
*Dorema aucheri*	Iran	hypoglycemic	[744]
*Eclipta alba*	Bangladesh, India (Ayurveda)	α-glucosidase inhibitor	[409,745,746]
*Elaeocarpus ganitrus*	India (Ayurveda), Nepal	antidiabetic	[747]
*Eleutherine palmifolia*	Indonesia	hyperglycemic	[748]
*Emblica officinalis*	India (Ayurveda), Bangladesh	antidiabetic	[89,409,749]
*Enhydra fluctuans*	India	antidiabetic	[750]
*Eremurus persicus*	Iran	antidiabetic	[751]
*Erigeron breviscapus*	China	antidiabetic	[752]
*Eryngium creticum*	Jordan	antidiabetic	[753]
*Eucommia ulmoides*	China, Japan, Korea	antidiabetic	[754]
*Eulophia herbacea*	Bangladesh	antidiabetic	[755]
*Fagonia cretica*	Pakistan	antidiabetic	[143,756]
*Fagopyrum cymosum*	China	hypoglycemic	[109]
*Feronia limonia*	India	antidiabetic	[757]
*Foeniculum vulgare*	Sudan, Iran, Portugal	antidiabetic	[154,758,759]
*Gloriosa superba*	India (Ayurveda)	antidiabetic	[760]
*Glycosmis pentaphylla*	Siddha, India (Ayurveda)	antidiabetic	[761]
*Gmelina arborea*	India, Sri Lanka	antidiabetic	[762,763]
*Gymnema sylvestre*	Ayurveda, Pakistan, Southeast Asia	hypoglycemic and antihyperglycemic	[356,764,765,766,767]
*Gynostemma pentaphyllum*	China, Vietnam	hypoglycemic	[768,769,770]
*Helianthus tuberosus*	Turkey	hypoglycemic	[176]
*Hemidesmus indicus*	India (Ayurveda)	antidiabetic	[771]
*Heritiera fomes*	India	antidiabetic	[772]
*Hippophae rhamnoides*	China	antidiabetic	[773]
*Hordeum vulgare*	Iran	antidiabetic	[774]
*Houttuynia cordata*	Japan	antidiabetic	[775]
*Ichnocarpus frutescens*	India (Ayurveda)	antidiabetic	[776]
*Imperata cylindrica*	India (Ayurveda)	antidiabetic	[777]
*Ixeris dentata*	Korea, Japan, and China	antidiabetic	[778]
*Juglans regia*	Iran, Algeria, Turkey, Austria	hypoglycemic	[779,780,781,782,783]
*Kaempferia parviflora*	Thailand	antidiabetic	[784]
*Kalopanax pictus*	Korea	antidiabetic	[785]
*Kickxia ramosissima*	Pakistan	antidiabetic	[786]
*Korthalsella japonica*	Korea	antidiabetic	[787]
*Lagenaria sicereria*	Mauritius, India (Ayurveda)	antihyperglycemic	[186,788,789]
*Lagerstroemia speciosa*	Philippines	hypoglycemic, α-glucosidase inhibitor	[790,791,792]
*Lannea coromandelica*	Bangladesh	antidiabetic	[793]
*Lactuca gracilis*	India	antidiabetic	[125]
*Leonurus sibiricus*	Mongolia	antidiabetic	[794]
*Leptospermum flavescens*	Malaysia	antidiabetic	[795]
*Linum usitatisumum*	India (Ayurveda)	α-amylase inhibitor	[128]
*Litchi chinensis*	Indonesia	antidiabetic	[796]
*Lycopus lucidus*	China (TCM), Korea	α-amylase inhibitor	[646,797]
*Macrotyloma uniflorum*	Asia, Africa	antidiabetic	[798]
*Magnolia officinalis*	China, Japan	antidiabetic	[799]
*Mahonia bealei*	China	antidiabetic	[800]
*Medicago sativa*	China	antidiabetic	[801]
*Meyna laxiflora*	India	antidiabetic	[802]
*Mezzetia parviflora*	Indonesia	antidiabetic	[803]
*Millingtonia hortensis*	India	antidiabetic	[125]
*Mitragyna speciosa*	Malaysia, Thailand, Southeast Asia	antidiabetic	[804]
*Mukia maderaspatana*	India (Ayurveda, Siddha)	antidiabetic	[805]
*Murdannia loriformis*	China	antidiabetic	[806]
*Myrica rubra*	China	antidiabetic	[807]
*Nelumbo nucifera*	India (Ayurveda), China (TCM), Southeast Asia	α-glucosidase, α-amylase inhibitor, hypoglycemic	[140,356,808,809]
*Neolamarckia cadamba*	Bangladesh	antidiabetic	[810]
*Nicotiana plumbaginifolia*	India	antidiabetic	[151]
*Nigella sativa*	Algeria, India (Ayurveda, Siddha, Unani), Pakistan, Morocco, Middle East, Mediterranean, North Africa	antidiabetic	[174,766,811,812,813,814,815,816]
*Nycantus arbor-tristis*	India (Ayurveda), Sri Lanka	hypoglycemic	[117]
*Nypa fruticans*	Malaysia	antidiabetic	[817]
*Odina wodier*	India	antidiabetic	[818]
*Ophiopogon japonicus*	China, Japan, Southeast Asia	antidiabetic	[181,819]
*Oreocnide integrifolia*	India	antidiabetic	[820]
*Oroxylum indicum*	Bangladesh, India (Ayurveda)	antidiabetic	[133,821]
*Paronychia argentea*	Jordan	hypoglycemic	[352,553]
*Pavonia zeylanica*	India (Ayurveda)	antidiabetic	[682]
*Pergularia daemia*	India (Ayurveda)	antidiabetic	[822]
*Persea americana*	Togo, Tanzania, Trinidad and Tobago, Central America, India (Ayurveda), Nigeria	antidiabetic	[180,188,189,438,823,824]
*Peucedanum praeruptorum*	India (Ayurveda), China	antidiabetic	[825]
*Phaseolus vulgaris*	Jordan	antihyperglycemic	[175,258]
*Phlomis armeniaca*	Turkey	α-amylase and an α-glucosidase inhibitor	[525]
*Phoenix dactylifera*	Jordan, India (Ayurveda), Pakistan, Egypt	antidiabetic	[258,826,827,828]
*Phragmanthera austroarabica*	Saudi Arabia	antidiabetic	[829]
*Phyllostachys edulis*	China	antidiabetic	[830]
*Pilea microphylla*	China	antidiabetic	[831]
*Pimpinella tirupatiensis*	Turkey, China, Korea, Iran, Egypt, Palestine, Lebanon, Europe	antidiabetic	[832,833]
*Pisonia grandis*	India	antidiabetic	[834]
*Platycodon grandiflorum*	Korea	antidiabetic	[835]
*Pluchea indica*	Indonesia	α-glucosidase inhibitor	[836]
*Plumbago zeylanica*	India	antidiabetic	[151]
*Polyalthia longifolia*	India	antidiabetic	[837]
*Polygonatum sibiricum*	China	antidiabetic	[181]
*Pongamia pinnata*	India (Ayurveda)	antihyperglycemic	[838,839]
*Poria cocos*	China	antidiabetic	[840]
*Portulaca oleracea*	Trinidad and Tobago, India (Ayurveda), Algeria, Iran, China (TCM), Mexico	hypoglycemic	[189,841,842,843,844,845,846]
*Premna integrifolia*	India (Ayurveda)	hypoglycemic	[113]
*Pseuderanthemum palatiferum*	Vietnam, Thailand	hypoglycemic	[847]
*Psoralea corylifolia*	India (Ayurveda)	antidiabetic	[848]
*Punica granatum*	India (Ayurveda, unani)	antidiabetic	[849,850,851,852]
*Raphanus sativus*	Iran, China	antidiabetic	[853,854]
*Rauwolfia serpentina*	Thailand	hypoglycemic	[433]
*Rehmannia glutinosa*	China, Korea	antidiabetic	[855,856]
*Retama raetam*	Saudi Arabia	antihyperglycemic	[857]
*Rhodamnia cinerea*	Malaysia	antidiabetic	[858]
*Roscoea purpurea*	Nepal	antidiabetic	[859]
*Rosmarinus officinalis*	Algeria, Jordan, Turkey	antidiabetic	[174,860,861]
*Roylea cinerea*	India	antidiabetic	[862]
*Rubia cordifolia*	India	antidiabetic	[863]
*Saccharum spontaneum*	India	antidiabetic	[125]
*Salicornia herbacea*	Korea	antidiabetic	[864]
*Sanguis draxonis*	China	antidiabetic	[865]
*Sasa borealis*	Korea	antidiabetic	[866]
*Schisandra chinensis*	China	antidiabetic	[181]
*Schizonepeta tenuifolia*	Korea	antidiabetic	[867]
*Securigera securidaca*	Iran	antidiabetic	[868]
*Sesbenia aegyptiaca*	India (Ayurveda)	hypoglycemic	[113]
*Siraitia grosvenori*	China	antidiabetic	[869]
*Sphaeranthus indicus*	India	antidiabetic	[870]
*Stevia rebaudiana*	India, Paraguay, Brazil, south America	antidiabetic	[871,872,873]
*Swietenia macrophylla*	Malaysia	antidiabetic	[874]
*Tamarindus indica*	India (Ayurveda), Trinidad and Tobago, Africa	α amylase inhibitor	[189,234,875]
*Tecoma stans*	Jordan, Central America, Egypt, Mexico	α-glucosidase inhibitor	[145,258,438,876]
*Tephrosia purpurea*	India (Ayurveda)	antihyperglycemic	[877,878]
*Thespesia populnea*	India (Ayurveda)	antihyperglycemic and hypoglycemic	[879]
*Tithonia diversifolia*	Costa Rica, Democratic Republic of Congo, Kenya, Nigeria, Mexico, the Philippines, São Tomé and Príncipe, Taiwan, Uganda, Venezuela	antidiabetic	[880]
*Toona sinensis*	China	antidiabetic	[881]
*Tragia involucrata*	India (Ayurveda)	antidiabetic	[882]
*Trichosanthis kirilowii*	China	antidiabetic	[181]
*Trigonella foenum-graecum*	Iran, Turkey, Algeria, Bangladesh, Pakistan, Morocco, Algeria, Mediterranean, China, India (Ayurveda)	antidiabetic, α-amylase inhibitor, antihyperlipidemic effect, hypoglycemic	[50,76,128,129,174,181,651,766,767,813,883,884,885,886,887,888,889]
*Varthemia iphionoides*	Jordan	antidiabetic	[753]
*Vinca major*	South Africa	antidiabetic	[441]
*Viola odorata*	India	antidiabetic	[151]
*Wedelia trilobata*	South America, China, Japan, India	antidiabetic	[890]

**Table 3:** Plant extracts with antidiabetic potential.

Species	Extract	Part of the Plant	Dosage (mg/kg)	Experimental Model	Induction of Diabetes	Reference
*Acacia arabica*	chloroform	bark	250, 500	male Wistar rats and albino mice	alloxan	[891]
	chloroform	bark	100, 200	female albino rats	streptozotocin	[892]
*Achyranthes rubrofusca*	aqueous and ethanolic	leaves	200	rats	alloxan	[893]
*Albizzia lebbeck*	methanol/dichloro-methane	stem bark	100, 200, 300, 400	male albino Wistar rats	streptozotocin	[894]
	methanolic	bark	200, 350, 620	female Sprague–Dawley rats	streptozotocin-nicotinamide	[895]
*Aloe vera*	aqueous	leaves	130	swiss albino mice	streptozotocin	[896]
	ethanolic	leaves	300	male albino Wistar rats	streptozotocin	[897]
*Amaranthus tricolor*	methanolic	whole plant	50, 100, 200, 400	male swiss albino mice	glucose-induced hyperglycemia	[898]
*Anacardium occidentale*	aqueous	leaves	175	male albino Wistar rats	streptozotocin	[899]
	methanolic	leaves	100	female albino mice	streptozotocin	[900]
*Azadirachta indica*	ethanolic	leaves	200	adult rabbits	alloxan	[901]
*Barleria prionitis*	ethanolic	leaves and root	200	adult albino rats	alloxan	[902]
*Bauhinia thoningii*	aqueous	leaves	500	Wistar albino rats	alloxan	[903]
*Caesalpinia ferrea*	aqueous	stem bark	300, 450	male Wistar rats	streptozotocin	[904]
*Camellia sinensis*	crude tea	leaves	0.5 mL/day	male albino mice	streptozotocin	[905]
*Casearia esculenta* Roxb	aqueous	root	200, 300	male albino Wistar rats	streptozotocin	[906]
*Cassia fistula*	ethanolic	stem bark	250, 500	Wistar rats	alloxan	[907]
*Cassia grandis*	aqueous and ethanolic	stem	150	male albino Wistar rats	alloxan	[908]
*Catharanthus roseus*	dichloromethane-methanol	leaves and twigs	500	male Sprague–Dawley rats	streptozotocin	[909]
	ethanolic	leaves	100, 200	male Wistar rats	streptozotocin	[711]
*Cecropia pachystachya*	methanolic	leaves	80	male Wistar rats	alloxan	[910]
*Ceriops decandra*	ethanolic	leaves	30, 60, 120	male albino Wistar rats	alloxan	[911]
*Chiliadenus iphionoides*	ethanolic	aerial parts	1000	male and female diabetes-prone *Psammomys obesus*	-	[912]
*Cinnamomum cassia*	ethanolic	bark	200, 300	male Kunming mice	streptozotocin	[913]
*Cinnamomum japonica*	ethanolic	bark	200, 300	male Kunming mice	streptozotocin	[913]
*Citrullus colocynthis*	aqueous	root	2000	male and female Wistar rats and Swiss albino mice	alloxan	[914]
	aqueous	seed	1, 2 mL/kg	male Wistar albino rats	alloxan	[915]
*Coscinium fenestratum*	ethanolic	stem	250	male albino Wistar rats	streptozotocin-nicotinamide	[916]
*Eucalyptus citriodora*	aqueous	leaves	250, 500	albino rats	alloxan	[917]
*Gymnema sylvestre*	ethanolic	leaves	100	male Sprague–Dawley rats	streptozotocin	[918]
*Heinsia crinata*	ethanolic	leaves	450–1350	rats	alloxan	[919]
*Helicteres isora*	butanol and aqueous ethanol	roots	250	male Wistar rats	alloxan	[920]
*Momordica charantia*	aqueous	pulp	13.33 g pulp/kg	male albino Wistar rats	alloxan	[921]
	ethanolic	fruit	200	adult rabbits	alloxan	[901]
	ethanolic	fruit	400	male Sprague–Dawley rats	streptozotocin	[922]
*Moringa oleifera*	methanolic	pod	150, 300	Wistar albino rats	streptozotocin	[923]
	-	leaves	50	male Sprague–Dawley rats	alloxan	[924]
*Murraya koenigii*	aqueous	leaves	200, 300, 400	male albino rabbits	alloxan	[458]
	ethanolic	leaves	100, 250	male albino Swiss mice	dexamethasone	[925]
*Opuntia ficus-indica*	petroleum ether	stems	200	male ICR mice	streptozotocin	[926]
*Origanum vulgare*	methanolic	leaves	5	male C57BL/6 mice	streptozotocin	[927]
*Passiflora nitida*	hydro-ethanolic	leaves	50	female Wistar rats	streptozotocin	[928]
*Paspalum scrobiculatum*	aqueous and ethanolic	grains	250, 500	male Wistar albino rats	alloxan	[929]
*Persea americana*	hydro-alcoholic	leaves	150, 300	male Wistar rats	streptozotocin	[930]
	aqueous	seed	20, 30, 40 g/L	male Wistar albino rats	alloxan	[931]
*Phoenix dactylifera*	ethanolic	leaves	50-400	male Wistar rats	alloxan	[932]
*Phyllanthus niruri*	aqueous	leaves	200, 400	male Wistar rats	streptozotocin-nicotinamide	[934]
*Phyllanthus simplex*	petroleum ether, ethyl acetate, methanol and water fraction		100–400	rats	alloxan	[935]
*Picralima nitida*	methanolic	steam bark and leaves	75, 150, 300	Wistar rats	streptozotocin	[936]
*Piper longum*	aqueous	root	200, 300, 400	male Wistar albino rats	streptozotocin	[937]
*Sonchus oleraceus*	hydro-alcoholic	whole plant	75, 150, 300	Wistar rats	streptozotocin	[936]
*Syzygium jambolana*	ethanolic	seed	200	adult rabbits	alloxan	[901]
*Tamarindus indica*	ethanolic	stem bark	250, 500	Wistar rats	alloxan	[907]
	ethanolic	seed coat	500	Wistar albino rats	alloxan	[938]
*Terminalia chebula*	chloroform	seed	100, 200, 300	male Sprague–Dawley rats	streptozotin	[939]
*Terminalia catappa*	petroleum ether, methanol and aqueous	fruit	68, 40, 42	Wistar albino rats and mice	alloxan	[940]
*Trigonella foenum-graecum*	ethanolic	seed	100, 500, 1000, 2000	male Wistar albino rats	alloxan	[941]
	hydro-alcoholic	seed	500, 1000, 2000	Sprague–Dawley rats	alloxan	[942]
*Vaccinium arctostaphylos*	ethanolic	fruit	200, 400	male Wistar rats	alloxan	[943]
*Vernonia amygdalina*	aqueous	leaves	100	Wistar albino rats	alloxan	[944]
*Witheringia solanacea*	aqueous	leaves	500, 1000	male Sprague–Dawley rats	GTT	[945]
*Zaleya decandra*	ethanolic	roots	200	Wistar albino rats	alloxan	[946]
*Zizyphus mauritiana*	petroleum ether, chloroform, acetone, ethanol and aqueous	fruit	200, 400	female Wistar rats	alloxan	[947]

* unless otherwise noted, GTT glucose tolerance test; ICR Institute of Cancer Research.

**Table 4:** Sources, structure, and target of some potential antidiabetic phytochemicals.

Compound	Sources	Structure	Target	Reference
Baicalein	*Oroxylum indicum*, *Scutellaria baicalensis*	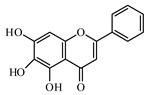	mitigates renal oxidative stress, suppresses activation of NF-κB, decreases expression of iNOS and TGF-β1, ameliorates structural changes in renal tissues, and normalizes the levels of serum proinflammatory cytokines and liver function enzymes	[953,987]
Berberine	*Argemone mexicana*, *Berberis aquifolium*, *Berberis aristata*, *Berberis vulgaris*, *Coptis chinensis*, *Eschscholzia californica*, *Hydrastis canadensis*, *Tinospora cordifolia*, *Xanthorhiza simplicissima*, *Phellodendron amurense*	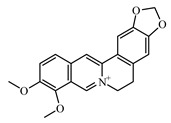	regulates glucose and lipid metabolism	[1041,1042]
Boldine	*Peumus boldus*	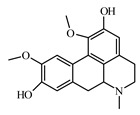	reduces overproduction of reactive oxygen species by inhibiting Ang II-stimulated BMP4 expression	[953,954]
Boswellic acids	the oleo gum resin from the trees of different *Boswellia* species (*Boswellia serrata*, *Boswellia carteri*)	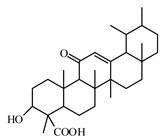	for the prophylaxis and/or treatment of damage to and/or inflammation of the islets of langerhans; stimulates β cells to release more insulin	[990,991]
Butein	*Toxicodendron vernicifluum*, *Dalbergia odorifera*, *Cyclopia subternata*, *Semecarpus anacardium*, *Creopsis tungtoria*	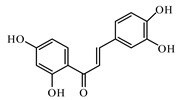	inhibits central NF-κB signaling and improves glucose homeostasis	[1016]
Catechins (catechin, epicatechin and epigallocatechin gallate (EGCG))	tea and cocoa, *Camellia sinensis*, *Theobroma cacao*	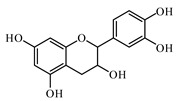	antioxidative; by protective effects against oxidative damage; by modification of oxidative stress; reduces lipid peroxidation by enhancing the SOD, GST, and CAT activities	[1043,1044]
Celastrol	*Tripterygium wilfordii*, *Celastrus orbiculatus*, *Celastrus aculeatus*, *Celastrus reglii*, *Celastrus scandens*	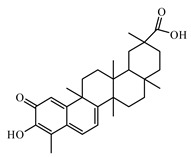	protective effects on diabetic liver injury via TLR4/MyD88/NF-*k*B signaling pathway in T2DM; suppresses obesity process via increase in antioxidant capacity and improves lipid metabolism; an NF-κB inhibitor; improves insulin resistance and attenuates renal injury	[992,993,994]
Chlorogenic acid	in many varieties of plant species	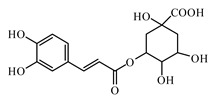	stimulates glucose transport in skeletal muscle via AMPK activation; effects on hepatic glucose release and glycemia	[1025,1026,1027]
Chrysin	*Passiflora caerulea*, *Passiflora incarnata*, *Oroxylum indicum*	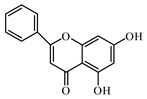	suppresses transforming growth factor-beta (TGF-β), fibronectin, and collagen-IV protein expressions in renal tissues; reduces the serum levels of pro-inflammatory cytokines, interleukin-1beta (IL-1β), and IL-6	[953,985]
Curcumin	*Zingiberaceae* plants, *Curcuma longa*	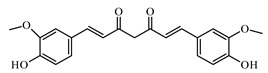	blood glucose-lowering effect; lowers glycosylated hemoglobin levels	[1017,1018,1019]
Ellagic acid	in fruits (pomegranates, persimmon, raspberries, black raspberries, strawberries, peach, plums), nuts (walnuts, almonds), vegetables, wine	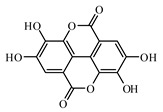	by the action on β cells of the pancreas that stimulates insulin secretion and decreases glucose intolerance; possesses superior antioxidant properties and genotoxicitypreventive; inhibits a-amylase activity; reduces hyperglycemia and insulin resistance in T2DM	[1028,1029,1030]
Embelin	*Embelia ribes*, *Lysimachia punctata*, *Lysimachia erythrorhiza*	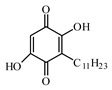	reduces the elevated plasma glucose, glycosylated hemoglobin, and pro-inflammatory mediators	[1031,1032]
Erianin	*Dendrobium chrysotoxum*	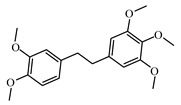	inhibits high glucose-induced retinal angiogenesis via blocking ERK1/2-regulated HIF-1α-VEGF/VEGFR2 signaling pathway	[1033]
Fisetin	*Acacia greggii*, *Acacia berlandieri*, *Gleditschia triacanthow*, *Butea fronds*, *Gleditsia triacanthos*, *Quebracho colorado*, *Rhus cotinus*, *Rhus vemiciflua* *Cotinus coggygria*, *Callitropsis* *Nootkatensis*	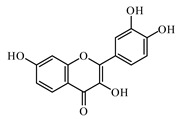	improves glucose homeostasis through the inhibition of gluconeogenic enzymes; increases the level and activity of glyoxalase 1; significantly reduces blood glucose	[963,964,965]
Galactomannan gum	*Cyamopsis tetragonolobus Amorphophallus konjac*	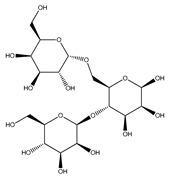	delays the rate of glucose absorption and thereby helps to reduce postprandial hyperglycemia	[1003,1004]
Gambogic acid	*Garcinia hanburyi.* *Garcinia indica*, *Garcinia cambogia*	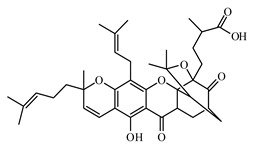	ameliorates diabetes-induced proliferative retinopathy through inhibition of the HIF-1α/VEGF expression via targeting the PI3K/AKT pathway	[1034]
Garcinol	*Garcinia* spp. plants (*Garcinia indica*)	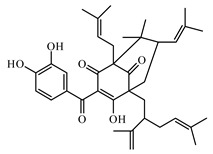	decreases plasma insulin, HOMA-β-cell functioning index, glycogen, high-density lipoprotein cholesterol, body weight, and antioxidant enzyme activities, viz. SOD, CAT, and glutathione; causes a significant reduction in elevated levels of blood glucose, glycosylated hemoglobin, and lipids	[1035,1036]
Honokiol	*Magnolia* plant spp. (*Magnolia officinalis*)	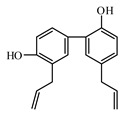	significantly increases phosphorylations of the IRβ and the downstream insulin signaling factors including AKT and ERK1/2; potential binding mode of honokiol to PTP1B; protects pancreatic β cells against high glucose and intermittent hypoxia-induced injury by activating the Nrf2/ARE pathway	[1037,1038]
Kaempferol	in a variety of plants and plant-derived foods	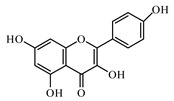	promotes insulin sensitivity and preserves pancreatic β-cell mass	[966]
Lupanine	*Lupinus* species (*Lupinus perennis)*	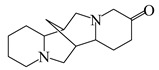	enhances insulin secretion; improves glucose homeostasis by influencing KATP channels and insulin gene	[955]
Luteolin	Lamiaceae plant family	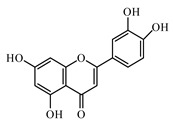	diabetic nephropathy; ameliorates cardiac failure in T1DM cardiomyopathy	[967,968]
Indole-3-Carbinol	in cruciferous vegetables	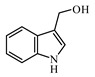	increases the antioxidant-scavenging action by increasing levels of SOD, CAT, GPx, vitamin C, vitamin E, and glutathione	[1023,1024]
Inulin	the *Helianthus tuberosus* tubers contain 75 to 80% of carbohydrates in the form of inulin	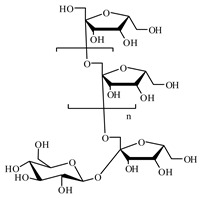	acts as a biogenetic factor for the development of natural intestinal microflora after dysbacteriosis; in the modulation of blood metabolites and liver enzymes	[1005,1006]
Morin	*Morus alba*, *Maclura pomifera*, *Psidium guajava*, *Chlorophora tinctoria*, *Prunus dulcis*, *Maclura tinctoria*, *Castanea sativa*	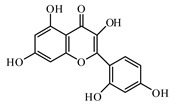	as an activator and sensitizer of the insulin receptor stimulating the metabolic pathways; rescues endothelial dysfunction in a diabetic mouse model by activating the Akt/eNOS pathway; downregulation of the miR-29a level; attenuates ER stress throughout the downregulation of the PERK-eIF2α-ATF4 pathway by interacting with the PERK protein	[975,976]
Naringenin	Grapefruit (*Citrus* × *paradisi*)	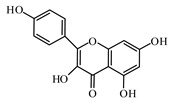	attenuates diabetic nephropathy via its anti-inflammatory and anti-fibrotic activities	[953,969]
Neferine	*Nelumbo nucifera*	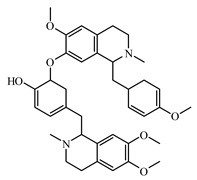	reduces expression of CCL5 and CCR5 mRNA in the superior cervical ganglion of T2D; prevents hyperglycemia-induced endothelial cell apoptosis through suppressing the OS/Akt/NF-κB signal	[953,957]
Oxymatrine	*Sophora flavescens*	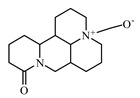	prevents oxidative stress and reduces the contents of renal advanced glycation end products, transforming growth factor-β1, connective tissue growth factor, and inflammatory cytokines in diabetic rats	[953,958]
Piceatannol	in a variety of plant sources (grapes, rhubarb, peanuts, sugarcane, white tea) and in the seeds of *Passiflora edulis*	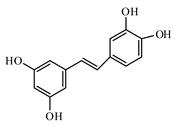	lowers the blood glucose level; promotes glucose uptake through glucose transporter 4 translocation to the plasma membrane in L6 myocytes; and suppresses blood glucose levels in T2DM	[1008,1009]
Piperine	*Piper* species (*Piper nigrum*, *Piper longum*)	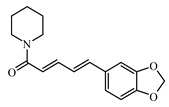	bio-enhancing effect of piperine with metformin in lowering blood glucose levels; blood glucose-lowering effect	[959,1045]
Quercetin	in many fruits, vegetables, leaves, grains	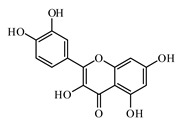	decreases the cell percentages of G(0)/G(1) phase, Smad 2/3 expression, laminin and type IV collagen, and TGF-β(1) mRNA level; activates the Akt/cAMP response element-binding protein pathway	[970,971]
Resveratrol	wine and grape (*Vitis vinifera*) juice, peanuts (*Arachis hypogaea*), pistachios (*Pistacia vera*), blueberries (*Vaccinium corymbosum*)	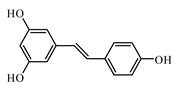	decreases blood insulin levels; reduces adiposity, changes in gene expression, and changes in the activities of some enzymes; enhances GLUT-4 translocation; activates SIRT1 and AMPK; affects insulin secretion and blood insulin concentration; reduces blood insulin; diabetes-related metabolic changes via activation of AMP-activated protein kinase	[1046,1047,1048,1049]
Rutin	present in certain fruits and vegetables	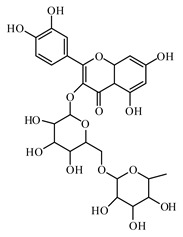	improves glucose homeostasis by altering glycolytic and gluconeogenic enzymes; involvement of GLUT-4 in the stimulatory effect on glucose uptake; potentiates insulin receptor kinase to enhance insulin-dependent glucose transporter 4 translocation	[972,973,974]
Sanguinarine	*Sanguinaria canadensis*	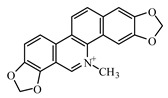	was targets and candidate agent for T2DM treatment with a computational bioinformatics approach	[960]
Silymarin	the milk thistle plant (*Silybum marianum*)	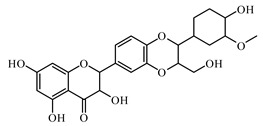	reduction in levels of blood glucose, glycosylated hemoglobin, urine volume, serum creatinine, serum uric acid, and urine albumin; nephroprotective effects in T2DM; ameliorates diabetic cardiomyopathy through the inhibition of TGF-β1/Smad signaling	[953,982]
Tocotrienol	in a wide variety of plants; *Bixa orellana*, *Zea mays*, *Garcinia mangostana*, *Elaeis guineensis*, *Hevea brasiliensis*	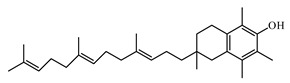	reduced the high-sensitivity C-reactive protein in a group of patients with T2DM; involved in the NF-κB signaling pathway, oxidative-nitrosative stress, and inflammatory cascade in an experimental model	[1021,1022]
Triptolide	*Tripterygium wilfordii*	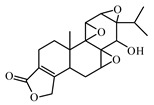	levels of phosphorylated protein kinase B and phosphorylated inhibitor of kappa B in splenocytes were reduced, and caspases 3, 8, and 9 were increased; diabetic nephropathy; triptolide treatment, accompanied with alleviated glomerular hypertrophy and podocyte injury	[1001,1002]
Ursolic acid, ursolic acid stearoyl glucoside	*Calluna vulgaris*, *Crataegus laevigata*, *Eriobotrya japonica*, *Eugenia jambolana*, *Melissa officinalis*, *Mentha piperita*, *Ocimum sanctum*, *Rosmarinus officinalis*, *Thymus vulgaris* *Dracocephalum heterrophyllum*, *Hyssopus seravshanicus*	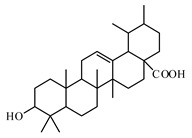	decreased hepatic glucose-6-phosphatase activity and increased glucokinase activity; reduced blood glucose levels; insulin secretagogue and insulinomimetic is mediated by cross-talk between calcium and kinases to regulate glucose balance	[1050,1051,1052]
Withanolides	*Withania somnifera* in plant sources from the Dioscoreaceae, Fabaceae, Lamiaceae, Myrtaceae, Taccaceae families	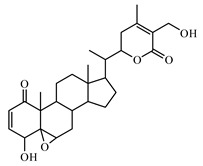	hypoglycaemic and hypolipidaemic activities	[1040]

AMPK 5′ AMP-activated protein kinase; ATF4 activating transcription factor 4;CAT catalase; eIF2α eukaryotic initiation factor 2 alpha; GPx glutathione peroxidase; GST glutathione S-transferase; KATP ATP-sensitive potassium; PERK endoplasmic reticulum kinase; SOD superoxide dismutase.
